# Lactate Efflux Inhibition by Syrosingopine/LOD Co‐Loaded Nanozyme for Synergetic Self‐Replenishing Catalytic Cancer Therapy and Immune Microenvironment Remodeling

**DOI:** 10.1002/advs.202300686

**Published:** 2023-06-29

**Authors:** Shengming Wu, Lehua Xu, Chenlong He, Peng Wang, Jingwen Qin, Fangfang Guo, Yilong Wang

**Affiliations:** ^1^ The Institute for Translational Nanomedicine Shanghai East Hospital The Institute for Biomedical Engineering and Nano Science School of Medicine Tongji University Shanghai 200092 P. R. China

**Keywords:** lactate efflux, nanocatalytic reaction, synergetic therapy, syrosingopine, tumor immunosuppressive microenvironment

## Abstract

An effective systemic mechanism regulates tumor development and progression; thus, a rational design in a one‐stone‐two‐birds strategy is meant for cancer treatment. Herein, a hollow Fe_3_O_4_ catalytic nanozyme carrier co‐loading lactate oxidase (LOD) and a clinically‐used hypotensor syrosingopine (Syr) are developed and delivered for synergetic cancer treatment by augmented self‐replenishing nanocatalytic reaction, integrated starvation therapy, and reactivating anti‐tumor immune microenvironment. The synergetic bio‐effects of this nanoplatform stemmed from the effective inhibition of lactate efflux through blocking the monocarboxylate transporters MCT1/MCT4 functions by the loaded Syr as a trigger. Sustainable production of hydrogen peroxide by catalyzation of the increasingly residual intracellular lactic acid by the co‐delivered LOD and intracellular acidification enabled the augmented self‐replenishing nanocatalytic reaction. Large amounts of produced reactive oxygen species (ROS) damaged mitochondria to inhibit oxidative phosphorylation as the substituted energy supply upon the hampered glycolysis pathway of tumor cells. Meanwhile, remodeling anti‐tumor immune microenvironment is implemented by pH gradient reversal, promoting the release of proinflammatory cytokines, restored effector T and NK cells, increased M1‐polarize tumor‐associated macrophages, and restriction of regulatory T cells. Thus, the biocompatible nanozyme platform achieved the synergy of chemodynamic/immuno/starvation therapies. This proof‐of‐concept study represents a promising candidate nanoplatform for synergetic cancer treatment.

## Graphical abstract

Cellular pH gradient reversal caused by lactate metabolism via glycolysis of tumor cells sets a big obstacle to efficient nanotherapeutics‐based cancer therapy. To solve this problem, hollow Fe_3_O_4_ nanozyme based biocompatible dual‐drug carrying nanoplatform is developed to achieve synergy of augmented chemodynamic/immuno/integrate starvation therapies through lactate efflux inhibition by a delivered clinically used drug.

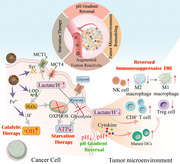

## 1 Introduction

A tumor develops an effective systemic mechanism, which enables its fast growth, migration, invasion, immune evasion, and distant metastases. This is typically reflected by the secretion of a large amount of lactic acid, which is a hallmark of abnormal energy metabolism in a hypoxia circumstance, known as “Warburg effect”.^[^ ^1^ ^]^ The resulting intracellular and extracellular reverse pH gradient is one of the direct manifestations of cancer biology that induces permissive intracellular surroundings for the tenacious viability of cancer cells and immunosuppressive tumor microenvironment (ISTME) for easier immune evasion.^[^ ^2^, ^3^, ^4^, ^5^, ^6^ ^]^ Recently, nanotherapeutics‐based therapies have received great attention in tackling lactic acid secretion for effective cancer treatment. There are two strategies to realize enhanced tumor inhibition upon targeting the reversed cellular pH gradient.^[^ ^7^ ^]^ One is the reduction of the degree of lactic acid secretion of tumor cells by specific reagents, such as siRNA and small molecular inhibitors, which leads to tumor depletion due to deterioration of both intracellular and extracellular surrounding for tumor progression.^[^ ^8^, ^9^ ^]^ However, the treatment efficacy is limited because of the metabolic adaption of tumor cells.^[^ ^10^ ^]^ The second strategy is combination therapy, which better solves this systemic disease via synergistic anti‐tumor effects.^[^ ^11^ ^]^

In lactate/H^+^ transportation, monocarboxylic acid transporter (MCT) proteins, especially MCT1 and MCT4, play critical but different roles.^[^ ^12^ ^]^ MCT4 is highly expressed only in hypoxia and rising concentrations of intracellular lactic acid, contributing to enhanced lactic acid secretion.^[^ ^13^ ^]^ Thus, MCT4 has been frequently down‐regulated as a target for nanocarrier‐based tumor treatment. Cai et al. created a surface functionalized hollow mesoporous organosilica nanoplatform loaded with hydroxycamptothecin and siMCT‐4 for down‐regulating expression of MCT4 to inhibit lactic acid efflux, which reactivates anti‐tumor immune microenvironment with enhanced chemotherapy efficacy.^[^ ^14^ ^]^ Recent studies found that carcinoma cells can use lactic acid as an energy supply,^[^ ^15^ ^]^ so it is suggested to rapidly consume the residual intracellular lactic acid after its efflux is blocked. To this end, the emerging nanocatalytic therapy provides a powerful tool with good specificity to inhibit the tumor by producing reactive oxygen species (ROS) hydroxyl radicals upon fast consumption of the residual lactate.^[^ ^16^ ^]^ Shi et al. developed an amorphous iron oxide nanoparticle (NP) based RNAi as the nanotherapeutics with multi‐functionalities, including modulating the glycolysis pathway by silencing MCT4 to induce tumor cell acidosis and concurrently exacerbating oxidative stress in tumor cells via Fenton‐like reaction.^[^ ^17^ ^]^ Gao et al. reported that utilization of a pH‐sensitive nanotherapeutics loaded with MCT1 inhibitor AZD3965 for tumor‐targeted therapy by inhibition of monocarboxylic acid transporter‐1 (MCT1) and reversal of lactic acid‐induced tumor immunosuppression microenvironment.^[^ ^18^ ^]^

Catalytic therapy, an emerging specifically therapeutic modality, has been compromised by a shortage of catalytic raw materials.^[^ ^19^ ^]^ For this reason, researchers mainly converted lactic acid and glucose at the tumor site into hydrogen peroxide through cascade catalytic reaction, which has been proven an effective means to improve the effectiveness of catalytic therapy.^[^ ^20^ ^]^ More recently, Lin et al. designed a new combination therapy strategy involving self‐replenishing nanocatalytic reaction and reconstruction of the immune microenvironment and starvation therapy based on glucose oxidase‐loaded TME‐activated UCNPs‐based enzyme nanocatalysts with cascade ROS amplification.^[^ ^21^ ^]^ Moreover, Qu et al. developed a new therapeutic mode through catalyzing lactic acid to supplement hydrogen peroxide by lactate oxidase (LOD), which produces more hydroxyl radicals for catalytic therapy and enhances tumor chemotherapy.^[^ ^20a^ ^]^ Compared to the relatively low concentration of glucose in tumors (1–2 µmol g^−1^), the intratumoral level of lactic acid (LA, 5–20 µmol g^−1^) suggests its greater potential for efficient tumor therapy.^[^ ^22^ ^]^

Despite the progresses in establishing nanoplatforms for cancer treatment based on the turnover of the cellular pH gradient reverse and cascade combination therapy, it is still highly imperative to construct a nanocatalyst carrier integrating augmented catalytic reaction, starvation therapy, and immune microenvironment reconstruction via a hydrogen peroxide (H_2_O_2_) open source and reduced expenditure strategy based on more integrate and straightforward regulation of cellular pH gradient reverse. It has been reported that syrosingopine (Syr), a clinically‐used anti‐hypertensive drug, blocks the functions of both MCT1 and MCT4, thereby reversing the pH gradient of tumors with hypoxic and phenotypic heterogeneity.^[^ ^23^ ^]^ Thus, developing a biocompatible and TME‐responsive nanocarrier to co‐deliver Syr and LOD for overturning the reversed cellular pH gradient by inhibition of lactate efflux and catalyzing lactate to H_2_O_2_ as a powerful trigger‐inducing synergy of tumor therapy may be useful.

**Scheme 1:**
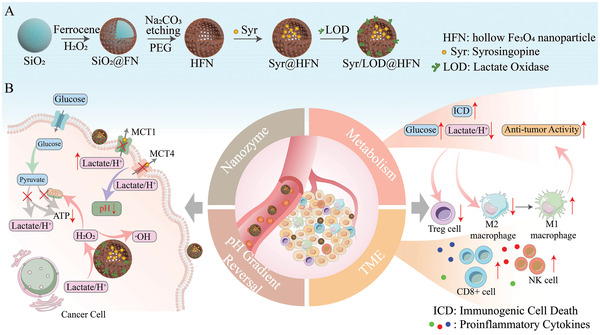
Schematic illustration of multifunctional nanoplatform Syr/LOD@HFN for enhanced tumor nanocatalytic‐starvation‐immuno therapy via inhibiting lactic acid efflux as a trigger.

Herein, we presented a hollow Fe_3_O_4_ nanoparticle‐based catalytic nanozyme carrier co‐loading Syr and LOD (Syr/LOD@HFN) for realizing the synergy of simultaneous self‐replenishing catalytic therapy, immune microenvironment remodeling, and integrated starvation therapy triggered by implementing reversal of cellular pH gradient. The nanoplatform with multifunctionality and availability of expansion based on one trigger aiming at the tumor biological cue was designed (as shown in **Scheme** **1**) with following advantages: (1) a clinically‐used hypotensor syrosingopine was delivered by a biocompatible and TME responsive nanocarrier to effectively block lactic acid secretion of tumor cells by depletion of both MCT1 and MCT4 functions; (2) augmented nanocatalytic reaction rate obtained from both intracellular acidification through reversal of cellular pH gradient and self‐replenishing supply of H_2_O_2_ via substantial transformation of lactate to H_2_O_2_ by co‐delivery of LOD with Syr; (3) facile exertion of integrate starvation tumor therapy covering ablation of cellular ATP supply from glycolysis and oxidative phosphorylation (OXPHOS) pathways; (4) facilitating immunogenic cell death (ICD) to enhance tumor inflammation to promote immune cell infiltration and the synergistic bio‐effects of remodeling of the TME induced by the inhibition of the tumor cells lactate/H^+^ efflux, including enhancement of the anticancer activity of macrophages, reactivation of effector T cells and NK cells, and inhibition of the immunosuppressive effect of regulatory T (Treg) cells. The versatile nanoplatform designed in a straight and facile way exhibited a one‐stone‐two‐birds effect without any dissipation of the function of each component.

## 2 Results and Discussion

### 2.1 Preparation and Characterization of Syr/LOD@HFN

**Figure 1:**
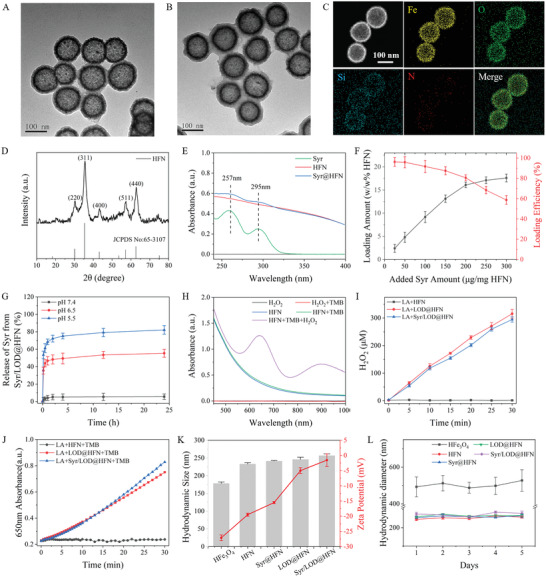
Fabrication and characterization of Syr/LOD@HFN. Representative TEM images of A) Syr@HFN and B) Syr/LOD@HFN. C) STEM image of the Syr/LOD@HFN, displaying the element distribution of Fe, O, Si, and N inside them. D) XRD pattern of the Syr/LOD@HFN. E) UV–vis absorption spectra of the free Syr, HFN, and Syr@HFN. F) Loading amount and loading efficiency of Syr by 1 mg of HFN at different Syr inputs. G) Syr release profiles of Syr/LOD@HFN under different pH conditions. H) UV–vis absorption spectra of the catalyzed oxidation of TMB (oxTMB) as catalyzed by 50 µg ml^−1^ HFN and 1 mm H_2_O_2_ in the reaction buffer (pH 6.5). I) Time‐dependent curves of concentrations of the H_2_O_2_ produced by catalyzing 2 mm of lactic acid by the respective HFN, LOD@HFN, and Syr/LOD@HFN. J) Plots of the time‐dependent absorbance change at 650 nm due to the catalyzed oxidation of TMB by the LOD@HFN and Syr/LOD@HFN. Reaction conditions: 50 µg ml^−1^ NPs, 200 µg ml^−1^ TMB, and 1 mm lactic acid at room temperature for 0.5 h in PBS (pH 6.5). K) Hydrodynamic diameters and Zeta potential (mV) distribution of the hollow Fe_3_O_4_ (HFe_3_O_4_), PEGylated hollow Fe_3_O_4_ (HFN), Syr@HFN, LOD@HFN, and Syr/LOD@HFN dispersed in deionized water respectively. L) Colloidal stability of the different nanoparticles dispersed in PBS (pH 7.4).

According to the synthetic method described in our previous study,^[^ ^24^ ^]^ 140 nm hollow Fe_3_O_4_ nanoparticles (HFe_3_O_4_) with monodispersity were synthesized and observed using transmission electron microscopy (TEM) (Figure S1, Supporting Information). In the X‐ray photoelectron spectroscopy (XPS) spectrum, the peaks located at 710.18 and 723.58 eV shown in Figure S2 (Supporting Information) are ascribed to the binding energy of Fe 2p_3/2_ and Fe 2p_1/2_ of Fe_3_O_4_ nanoparticles, respectively, which is consistent with the data of Fe_3_O_4_ reported in the literature.^[^ ^25^ ^]^ After PEGylation of the HFe_3_O_4_, HFN was obtained for dual‐drug loading. Then, Syr/LOD@HFN was prepared by mixing Syr and LOD with HFN, as shown in Scheme 1A. The typical morphologies of the obtained Syr@HFN and Syr/LOD@HFN were observed using TEM. It was found that the original uniform hollow structure of the HFN does not change during Syr and LOD loading (**Figure** **1**A,B). Subsequently, the morphology and elements feature of the Syr/LOD@HFN were further examined using elemental mapping on a high‐angle annular dark‐field scanning TEM (HAADF‐STEM). The as‐prepared Syr/LOD@HFN shows clear element signals of Fe, O, Si, and N (Figure 1C), which were also confirmed by recording their energy dispersive spectra (Figure S3, Supporting Information). Furthermore, the chemical composition of the as‐synthesized HFN was examined by X‐ray powder diffraction (XRD). As shown in Figure 1D, sharp diffraction peaks of the HFN are well indexed with (220), (311), (400), (511), and (440), indicating successful preparation of hollow Fe_3_O_4_ nanoparticles (JCPDS No: 65–3107).^[^ ^26^ ^]^

To examine the payload of the Syr by the nanocarriers, UV–vis absorption spectra of pure Syr, HFN and Syr@HFN are shown in Figure 1E. Obvious absorption peaks at ≈ 257 and 295 nm of the Syr@HFN were the same as that from pure Syr, indicating successful drug loading. Additionally, the loading amount of Syr by the HFN was calculated by a subtraction method based on the corresponding UV–vis absorption spectra (Figure S4, Supporting Information). Various masses of Syr ranging from 25 to 300 µg were used for optimization of the Syr loading based on 1 mg of HFN. With an increase of Syr input, the payload gradually rose to 18 w/w% (Figure 1F) due to the cavity structure and monodispersity of the HFN. Because continuing rising of Syr input did not result in an obvious increase in the loading capacity of Syr, the Syr to HFN mass ratio of 200 µg mg^−1^ was applied in the subsequent experiments to synthesize the Syr@HFN with a peak loading efficiency of > 80% (Figure 1F). Interestingly, the relative release of the Syr in the Syr@HFN was < 15% when being incubated in the medium at pH 7.4 within up to 24 h, while the value increased significantly at either pH 6.5 or 5.5. As shown in Figure S5 (Supporting Information), at pH 6.5, the Syr@HFN exhibited a robust release of > 40% of the loaded Syr within 10 min, and the cumulative release reached 69% for 24 h (Figure 1G). Furthermore, at pH 5.5, the cumulative release of the Syr reached up to 80% within 24 h. It is worth noting that the release profile of the Syr by the Syr/LOD@HFN was extremely similar to that from the Syr@HFN. Release of Syr by the nanozyme exhibited a weak acidic condition enhanced feature, which may benefit TME‐specific drug delivery and reduction of possible toxic side effects.^[^ ^27^ ^]^

In this study, the HFe_3_O_4_ nanoparticles, as one of the classical Fenton reaction nanozymes, efficiently converted H_2_O_2_ into highly toxic •OH at pH 6.5, which was manifested by a significant UV absorption peak at 650 nm derived from the oxidation of TMB to oxTMB (Figure 1H).^[^ ^20b^ ^]^ To testify successful preparation of the dual‐drug carrying system Syr/LOD@HFN, besides Syr, loading of the LOD by the Syr/LOD@HFN was conducted according to a previously reported process.^[^ ^8^, ^20^ ^]^ The loading capacity of LOD by the LOD@HFN and the Syr/LOD@HFN was 1.16±0.17 and 1.05±0.22 w/w%, respectively, which was calculated by measuring the LOD elution using BCA protein assay kit. LOD was supposed to adsorb on the surface of the nanocarriers through physical adsorption (hydrophobic interaction, Van Der Waals forces, electrostatic adsorption, etc.), like the formation of “protein corona” on nanoparticles’ surface.^[^ ^8^, ^28^ ^]^ LOD was also released in a pH‐dependent way, with the fastest release at pH 5.5 and a maximum release of ≈ 50% within 24 h (Figure S6, Supporting Information). The nanoparticles loaded with either the individual LOD or the dual‐drug exhibit efficient H_2_O_2_ conversion, catalyzing the production of 300 µm of H_2_O_2_ within 30 min (Figure 1I), which was much higher than the average concentration of the endogenous H_2_O_2_ in the tumor. After clarifying the successful transformation of lactic acid into H_2_O_2_ by the nanoparticles, the catalytic activity of the LOD@HFN and Syr/LOD@HFN in generating •OH from H_2_O_2_ were investigated via oxidation of TMB into oxTMB, which were testified by both UV–vis absorption of oxidized TMB at 650 nm and corresponding color change of the aqueous solution (Figure 1J; Figure S7, Supporting Information).

Figure 1K shows the evolution of the Zeta potential and hydrodynamic size of the nanocarriers dispersed in deionized water at each step during preparation of the Syr/LOD@HFN, indicating slowly rising diameters and rapid drop of the net negative surface potentials. These data gave indirect evidence of the successful loading of the dual drug. PEG molecules were used to modify HFe_3_O_4_ nanoparticles to obtain the HFN and improve the colloidal stability and biocompatibility of the drug‐loaded nanocarriers. The colloidal stability of the HFN and the drug‐loaded nanocarriers were examined when being dispersed in the PBS or FBS, proving the improved stability after PEG modification over the original hollow Fe_3_O_4_ nanoparticles (Figure 1L; Figure S8, Supporting Information).

Moreover, it was found that Syr/LOD@HFN gradually dissociated under weakly acidic conditions, probably facilitating the excretion of HFN from the body after realizing its functions (Figure S9, Supporting Information).^[^ ^29^ ^]^

### 2.2 Tumor Starvation Therapy via Braked Tumor Glycolysis and ROS‐Induced Mitochondria Dysfunction Upon Efficient Inhibition of the Lactic Acid Efflux by the Syr/LOD@HFN in vitro

Based on the preliminary investigation of physiochemical properties of the nanozyme platform, to further study the synergistic bio‐effects of dual‐drug co‐delivery nanocarriers in vitro, inhibition of lactic acid secretion and overturning cellular pH gradient reverse by Syr was investigated. B16‐F10, a mouse‐derived melanoma tumor cell line with strong aerobic glycolysis (Warburg phenotype) and high expression of MCT1 and MCT4,^[^ ^30^ ^]^ was used. As is well known, in solid tumors, lactic acid, as a product of high degree aerobic glycolysis, is excreted into TME through cellular membrane mediated by the MCT proteins, including MCT1 and MCT4. In this study, the function of these two MCT proteins was simultaneously hampered by the delivered Syr, leading to rapidly rising residual lactic acid inside tumor cells, which is a direct trigger for designed synergistic bio‐effects of the nanoplatform.

**Figure 2:**
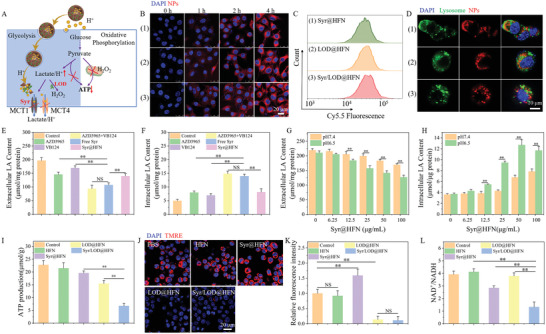
Syr/LOD@HFN inhibits tumor ATP supply. A) Schematic of Syr/LOD@HFN simultaneously cutting off glycolysis and oxidative phosphorylation of tumor cells. B) Confocal images and C) flow cytometry of B16‐F10 cells incubated with Cy5.5 labeled (1) Syr@HFN, (2) LOD@HFN, and (3) Syr/LOD@HFN for 0, 1, 2, 4 h. D) Confocal images of Cy5.5 labeled (1) Syr@HFN, (2) LOD@HFN and (3) Syr/LOD@HFN incubated with B16‐F10 cells for 4 h. The lysosomes were stained with LysoTracker Green. E) Extracellular and F) intracellular lactic acid content in the B16‐F10 cells treated with HFN, MCT1 inhibitor AZD3965, MCT4 inhibitor VB124, Free Syr and Syr@HFN for 6 h. G) Extracellular and H) intracellular lactic acid content in B16‐F10 cells treated with Syr@HFN at pH 6.5 or 7.4 for 6 h. I) ATP levels of the B16‐F10 cells after various treatments for 24 h. J) Confocal images of the mitochondrial membrane potential of B16‐F10 cells tested by Tetramethylrhodamine ethyl ester perchlorate (TMRE) after the different treatments and (K) corresponding statistical graph of fluorescence intensity. L) NAD+/NADH ratio of the B16‐F10 cells after NPs (100 µg ml^−1^, pH 6.5) treatment for 24 h. All scale bars = 20 µm. Data are present as mean ± SEM. (p‐Values were calculated by using unpaired *t*‐test, *n* = 3, ^*^ *p* < 0.05, ^**^ *p* < 0.01).

As shown in the scheme of **Figure** **2**A, a combination of the Syr and LOD were expected to possess a dual role in tuning the metabolism of the tumor cells, including both tackles of glycolysis due to the fast accumulation of the intracellular residual lactic acid, and induction of mitochondrial dysfunction by the produced ROS via nanocatalytic reaction of the platform with the help of the transformation of the lactic acid to H_2_O_2_ by the LOD. Among them, simultaneous inhibition of the MCT1 and MCT4 by the Syr has a critical role because the expression of MCT1 and MCT4 varies with changing intracellular lactic acid concentration.^[^ ^23^, ^31^ ^]^ Figure 2B presents the fluorescent images of internalization of the Cy5.5 labeled Syr@HFN, LOD@HFN, and Syr/LOD@HFN by the B16F10 cells at intervals of 0, 1, 2, 4 h, which implied successful uptake of nanocarriers after incubation for 1 h. Moreover, flow cytometry results showed no significant difference in endocytosis of the three kinds of nanoparticles by the B16‐F10 cells after 4 h incubation (Figure 2C). To further explore the transportation pathway of the nanoparticles after endocytosis, B16‐F10 cells lysosomes were labeled with Lysotracker‐Green dye after 1 h or 4 h of co‐incubation of the cells with three kinds of nanoparticles respectively: (1) Syr@HFN, (2) LOD@HFN, and (3) Syr/LOD@HFN (Figure 2D; Figure S10, Supporting Information). Co‐localization and departure of the nanoparticles (red) and lysosomes (green) were monitored by dynamic observation of the fluorescent signal from the individual nanoparticles and lysosomes and the overlap of their signals. Utmost separation of the red and green fluorescent signal observed after 4 h of incubation (as shown in Figure 2D) indicated that the nanoparticles transported to the cellular plasma via endocytosis and lysosomal escape.^[^ ^32^ ^]^ Subsequently, Mander's overlap coefficients (MOC) were calculated using the Image J software to quantify the lysosomal localization of three kinds of nanoparticles (Figure S10B, Supporting Information). The results showed that with an extension of the incubation time of the B16‐F10 cells and nanoparticles, more nanoparticles escaped from the lysosomes into the cytoplasm, and the MOC value decreased. Notably, the lysosomal escape ability of the Syr/LOD@HFN was higher than that of Syr@HFN but similar to the LOD@HFN group. The possible reason was that the LOD@HFN and Syr/LOD@HFN could catalyze the production of H_2_O_2_ from the lactic acid, which damaged the lysosome and achieved a more efficient lysosomal escape.^[^ ^32^ ^]^

To further assess the inhibition efficiency of lactate efflux by the Syr, the amount of intracellular and extracellular lactate was measured. After 6 h of free Syr treatment, lactate secretion was reduced by 45.6% in the B16‐F10 cells, whereas it was reduced by only 25.9% and 13.8% in the cells treated with the MCT1 inhibitor AZD3965 or the MCT4 inhibitor VB124 alone, respectively (Figure 2E).^[^ ^33^ ^]^ In contrast, the efficiency of lactate efflux inhibition increased to 52.4% after simultaneous treatment of cells with both MCT1 inhibitor AZD3965 and the MCT4 inhibitor VB124, which was comparable to the free Syr inhibition efficiency. The results of cellular lactate amount showed the same pattern; only the simultaneous inhibition of MCT1 and MCT4 could achieve a large accumulation of lactate in the cells (Figure 2F). The results suggested that simultaneous inhibition of MCT1/MCT4 is more effective in hampering the lactate efflux than mere inhibition of MCT1 or MCT4, which is consistent with the finding that most solid tumors express both MCT1 and MCT4.^[^ ^23^, ^34^ ^]^

As expected, treatment of the B16F10 cells by the Syr@HFN at pH 7.4 only inhibited 29% lactate efflux, which was reasonable because it is difficult for the loaded Syr in the nanocarriers to be effectively released and impact the cells in the weak alkaline medium (Figures 1G and 2E). Contrarily, lactate efflux inhibition by Syr@HFN was comparable to that of free Syr in a cell culture medium at pH 6.5 (Figure 2G). In terms of the intracellular amount of lactate, it was found that the amount of lactic acid accumulated inside the cells was increased by three times due to the released Syr (Figure 2H).

Increased residual lactate led to higher intracellular lactate concentration that promoted inhibition of end‐product LDH and, following a loss of NAD^+^ regenerating capacity,^[^ ^23^, ^35^ ^]^ and ultimately leading to the “starvation” induced death of tumor cells. Interestingly, the ATP content of B16‐F10 cells treated by the Syr@HFN slightly decreased (Figure 2I). It was supposed that the loss of ATP due to the inhibition of glycolysis of the tumor cells was compensated by their enhanced aerobic metabolism.^[^ ^23^, ^35^ ^]^ Analyses of the mitochondrial membrane potentially supported this hypothesis (Figure 2J,K). After treatment with the Syr@HFN, the mitochondrial membrane potential of the tumor cells increased by 60%, implying that the activated aerobic metabolism made up the energy gap. Furthermore, the ATP level of the B16‐F10 cells treated by the Syr/LOD@HFN was reduced by 70%, which was probably attributed to the mitochondrial dysfunction derived from the fast accumulation of the hydrogen peroxide catalyzed by the LOD (Figure 2I–K). Additionally, the changes in the intracellular NAD^+^ and NADH content also supported the assumption that neither inhibition of the lactate efflux by the Syr@HFN alone nor damage to the mitochondria by the LOD@HFN significantly altered NAD^+^ and NADH content, indicating that the NAD^+^/NADH cycle was well‐maintained, with only the Syr/LOD@HFN‐treated cells having NAD^+^ production blocked and significantly reducing its content (Figure 2L; Figure S11, Supporting Information). These results imply that the strategy of co‐delivery of Syr and LOD may pose a bigger potential in tumor starvation therapy over inhibition of glycolysis or OXPHOS alone due to the tumor adaptability.^[^ ^36^ ^]^

### 2.3 In Vitro Augmented Self‐Replenishing Nanocatalytic Tumor Inhibition based on Tuning of Reversed Cellular pH Gradient by the Syr/LOD@HFN

**Figure 3:**
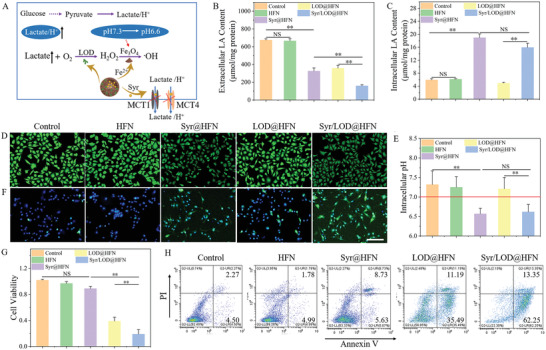
Syr/LOD@HFN reduces the cytoplasmic pH to speed up the catalytic reaction and realize the continuous supply of raw materials for catalytic reaction through cascade reaction. A) Schematic illustration of the Syr/LOD@HFN enhanced catalytic therapy. B) Extracellular and C) intracellular lactic acid content in the B16‐F10 cells upon various NPs (100 µg mL^−1^) treatment for 24 h (value was normalized based on 1 mg of total intracellular proteins measured by BCA assay kit). D) Fluorescent images of B16‐F10 cells showing the changes in the intracellular pH status with different treatments using BCECF‐AM. Weaker green fluorescence indicates a lower pH value. E) Intracellular pH after multiple treatments. F) Fluorescence images of 2”,7”‐Dichlorodihydrofluorescein diacetate (DCFH‐DA) stained B16‐F10 cells (525 nm) after various treatments for 1 h, respectively. Scale bar: 100 µm. G) Cell viability of B16‐F10 cells treated with multiple NPs (100 µg mL^−1^) for 24 h. H) Apoptosis of cells with different treatments assessed with AnnexinV‐FITC/PI by flow cytometry. Data are present as mean ± SEM. (p‐Values were calculated by using unpaired *t*‐test, *n* = 3, ^*^ *p* < 0.05, ^**^ *p* < 0.01).

With the developed nanocatalysts for tumor chemodynamic therapy, the problem of inappropriate pH value in the cytoplasm, the major site of catalytic reaction, has been attracting emerging attention.^[^ ^24^, ^37^, ^38^ ^]^ As shown in the scheme of **Figure** **3**A, the tumor cells usually undergo intense glycolysis to raise energy for rapid cell proliferation and produce a large amount of lactic acid. With continuous secretion of lactic acid from tumor cells mediated by MCT proteins and the existence of high concentrations of bicarbonate, the tumor cytoplasm was in a weak basic condition. However, when the Syr‐protein binding impedes transmembrane transportation of the lactic acid through MCT1/MCT4, existed reverse pH gradient between cellular membranes is overturned owing to an inhibition of the lactic acid efflux.^[^ ^23^ ^]^ To examine this process, the expression of the MCT1 and MCT4 proteins in the tumor cells under different conditions was analyzed. The results showed no significant change in the expression of the MCT1 and MCT4 in the B16‐F10 cells treated with various nanoparticles, which is because the Syr hardly decreases the expression of the MCT proteins but inhibits their function by the Syr‐MCT binding (Figure S12, Supporting Information).^[^ ^23^ ^]^ Subsequently, the extracellular content of the lactic acid secreted by the tumor cells in various conditions was analyzed. After 24 h of culture, the content of the lactic acid secreted by 1.0×10^4^ B16‐F10 cells into the medium was 675.1 µmol per 1 mg reference protein (total intracellular proteins measured by BCA assay kit), while the counterpart content decreased to ≈ 325.4 and 159.4 µmol per 1 mg protein after being treated by 100 µg mL^−1^ of Syr@HFN and Syr/LOD@HFN, respectively (Figure 3B). The extracellular lactate content of the cells with Syr/LOD@HFN treatment was significantly lower than that with Syr@HFN and LOD@HFN because of the combined lactate efflux inhibition and lactate depletion.

Subsequently, the amount of residual lactic acid in the tumor cells was analyzed. It was found that the amount of intracellular residual lactate in the Syr@HFN treatment group was 3.18‐fold that of the control group (Figure 3C). Despite consuming the residual lactate by the loaded LOD in the Syr/LOD@HFN, the intracellular lactate content was not significantly reduced than that of the Syr@HFN group because of the complex intracellular environment and continuous lactate production. An increase of the residual lactic acid induced the change of intracellular pH from 7.3 to 6.6, changing the pH condition from alkaline to acidic (Figure 3D,E; Figure S13, Supporting Information). UV–vis absorption spectra of the substance oxTMB obtained from oxidation of the TMB by the nanozymes at neutral or alkaline conditions indicated that the catalytic reactivity of nanozymes become extremely low and it hardly produced sufficient amount of •OH for tumor treatment (Figure S14, Supporting Information). However, the fluorescent images of the tumor cells with different treatments, as shown in Figure 3F, indicated much‐enhanced efficiency of the ROS production for the nanocatalytic therapy triggered by the biomimetic Syr/LOD@HFN nanoplatform via the cascade catalytic reactions. It is worth noting that the intracellular ROS content of the B16‐F10 cells treated by the non‐cytotoxic Syr@HFN also significantly increased, which was attributed to the enhanced ROS production by the mitochondria via the compensatory aerobic metabolism (Figure 3F). However, such mitochondrial ROS were less cytotoxic superoxide anion radicals and hardly exhibited obvious cell damage.^[^ ^38^ ^]^ These experimental results were consistent with the aforementioned result of the mitochondrial membrane potential measurement (Figure 2J). Corresponding results of cell viability assays indicated that the HFN hardly exhibited very limited cytotoxicity due to the inadequate concentration of H_2_O_2_ in the tumor cells and the weakly basic cytoplasm (Figure 3G). Meanwhile, the Syr@HFN exhibited negligible cytotoxicity, suggesting that it is difficult to display sufficient anti‐tumor efficacy even at an appropriate lower cytoplasmic pH condition but without a necessary H_2_O_2_ supply (Figure 3G). Moreover, the cytotoxicity was significantly compromised in the group of LOD@HFN compared to Syr/LOD@HFN, indicating the decisive role of Syr in producing sufficient toxic •OH via the Fenton reaction under the cytoplasmic weakly acidic conditions (Figure 3G). It was clear that adequate H2O2 and weak acidic circumstances were essential for efficient tumor catalytic therapy.^[^ ^19^ ^]^ Also, flow cytometry analyses of the tumor cells treated by the different nanoparticles further confirmed a highly effective catalytic anti‐tumor efficacy of the Syr/LOD@HFN (Figure 3H).

We summarized the biochemical pathway underlying the catalytic therapeutic bio‐effects of the nanoplatform Syr/LOD@HFN exerting on the tumor cells as follows: first, the MCT1/MCT4 proteins mediated lactate efflux was blocked by the carried syrosingopine for enhanced accumulation of the lactate inside the cell, with reduced cytoplasmic pH; second, co‐loaded LOD by the nanoplatform converted part of the intracellular residual lactate into the less toxic H_2_O_2_. Finally, Fe_3_O_4_ nanozyme catalyzed self‐replenishing H_2_O_2_ to the more toxic •OH through the Fenton reaction at a dramatically accelerated rate due to the improved cytoplasmic acidity. Meanwhile, the pathway of substitute OXPHOS as energy supply was partially compromised due to the influence of the toxic radicals on the mitochondrial function.

### 2.4 In Vivo Anti‐Tumor Effects of Synergistic Therapy by the Syr/LOD@HFN Nanozyme Platform

**Figure 4:**
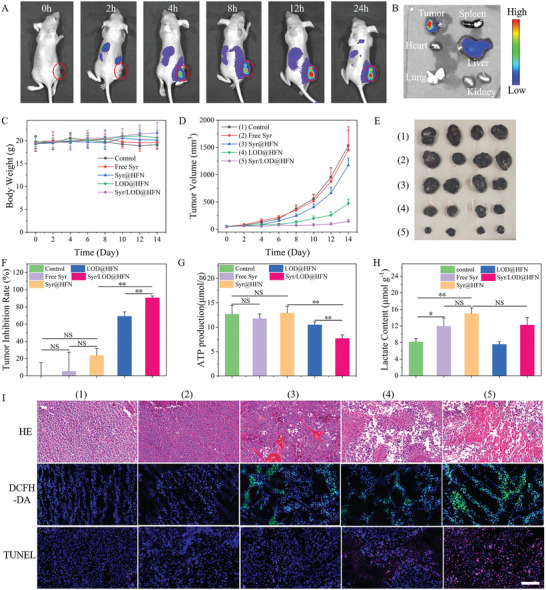
In vivo tumor catalytic therapy. A) In vivo fluorescence imaging of B16‐F10 tumor‐bearing nude mice demonstrating the accumulation of Cy5.5 labeled Syr/LOD@HFN at the tumor site over time. B) Biodistribution of NPs at various organs and tumor 24 h post‐injection. C) Average body weights of these B16‐F10 tumor‐bearing nude mice with various treatments as indicated (*n* = 4). D) Tumor growth curves, E) photograph of the tumor, F) tumor inhibition efficiency of these B16‐F10 tumor‐bearing nude mice with various treatments. G) ATP production and H) Lactate content of mouse tumors 24 h after drug injection. I) H&E staining for pathological changes, DCFH‐DA immunofluorescence staining for ROS, and TUNEL immunofluorescence staining for cellular apoptosis in tumor tissues from each group after a 14‐day therapeutic period. Scale bar: 100 µm. Data are present as mean ± SEM. (p‐Values were calculated by using unpaired *t*‐test, *n* = 4, ^*^ *p* < 0.05, ^**^ *p* < 0.01).

Next, in vivo anti‐tumor effects upon self‐replenishing and augmented nanocatalytic reactivity of the Syr/LOD@HFN platform were studied. First, the pharmacokinetic features of the Syr/LOD@HFN were carefully evaluated by tracking the fluorescence signal of the Cy5.5 labeled nanocarriers on tumor‐bearing nude mice models. The results showed that the tumors of nude mice with the intravenously injected Syr/LOD@HFN (concentration of Fe_3_O_4_: 20 mg kg^−1^) exhibited a gradual increase of the Cy5.5 fluorescence signals under the in vivo optical imaging system (IVIS), displaying the strongest fluorescent intensity at 12 h (**Figure** **4**A). Syr/LOD@HFN injection (after 24 h), the tumors showed the highest fluorescence intensity compared to other major organs, such as the liver, kidney, heart, spleen, and lung (Figure 4B). Besides the tumor, the liver also exhibited a strong fluorescence signal due to the fact that the hepatic reticuloendothelial system trapped a large number of nanocarriers.^[^ ^39^ ^]^

Next, we carefully compared the in vivo therapeutic efficacy of the Syr/LOD@HFN and some controls based on the B16‐F10 tumor‐bearing nude mice, which were randomly divided into five groups as bellow: (1) a control group with PBS only, (2) Free Syr, (3) Syr@HFN, (4) LOD@HFN, and (5) Syr/LOD@HFN. During 14 days of treatment, the nanocarriers were administrated on days 0, 3, 6, 9, and 12, and tumor volumes were measured on alternate days. During treatment, the body weights of the nude mice in the four therapeutic groups showed negligible differences compared to the control group (Figure 4C). In addition, hemolysis experiments showed that the dual‐drug loaded nanoparticles at various concentrations caused acceptable damage to the red blood cells, while no significant hemolysis was detected by co‐incubating cells with 100 µg ml^−1^ different nanoparticles for 1 h (Figure S15, Supporting Information). Moreover, hematoxylin and eosin (H&E) staining of the major organs of the nude mice further confirmed the biocompatibility of the nanozyme platform (Figure S16, Supporting Information).

Moreover, tumor growth curves showed that neither the free Syr nor the Syr@HFN inhibited tumor growth, with inhibiting efficiencies of only 5.3% and 23.5%, respectively (Figure 4D,E). Contrarily, the suppression efficiency of the tumor by the LOD@HFN reached 69.1% because they catalyzed the intracellular lactate to H_2_O_2_ with obvious cytotoxicity. More importantly, the inhibition efficiency of the tumor by the Syr/LOD@HFN was increased to 90.5%, proving that increased residual lactate accelerates the catalytic reaction accompanied by starvation therapy, which was an effective mean for the synergistic cancer treatment (Figure 4F; Figure S17, Supporting Information).

The coefficient of drug interaction (CDI) was used to calculate the interaction between two drugs and to quantify the synergistic effect between the tumor cell death inducers: CDI > 1 indicated antagonism, CDI = 1 indicated additivity and CDI < 1 indicated synergy; the smaller the CDI value, the higher the synergy. The interaction between Syr@HFN and LOD@HFN was synergistic, with a CDI coefficient of 0.4. This result suggested that Syr/LOD@HFN, which combines enhanced catalytic therapy and starvation therapy, is a highly efficient therapeutic nanoplatform.^[^ ^40^ ^]^

In this case, integrated starvation therapy was achieved by simultaneously inhibiting the aerobic metabolism OXPHOS and glycolysis through effective depletion of the ATP supply to the tumor cells in vivo. It was supported by the in vivo evidence that the ATP level inside the tumor treated by the Syr/LOD@HFN decreased by respective 39.3% and 18.0% to that of the tumor treated by the Syr@HFN and LOD@HFN (Figure 4G). The lactate amount in the tumor was examined to further assess lactate levels under the different treatments in vivo. Figure 4H shows that lactate amount in the group of Syr@HFN exhibited respective 83.9% and 25.6% increases over the counterpart tissue samples treated by the control and free Syr group, respectively, suggesting the efficient inhibition of the cellular lactic acid secretion by the nanocarrier delivered Syr. The resulting intracellular lactate accumulation was the cornerstone for the smooth operation of the treatment process. Moreover, H_2_O_2_ assay data showed that the H_2_O_2_ content experienced a substantial elevation after the Syr/LOD@HFN and LOD@HFN treatment, not only in the tumor stroma but also inside the tumor cells (Figure S18, Supporting Information). H_2_O_2_ was indispensable for efficient catalytic therapy as a raw material for catalytic reactions. Furthermore, it was noteworthy that the H_2_O_2_ content inside the tumor cells was > 10 times that in the tumor stroma, which indicates that the catalytic reaction mainly happened at the intracellular sites, and the ROS produced in the tumor stroma was not only in a relatively less level but also difficult to exert damaging effects on the cells due to its big distance from the organelles and nucleus. This also demonstrated the necessity of the strategies leading to lower intracellular pH considering the limited catalytic reaction rate in a weakly alkaline cytoplasm even with abundant H_2_O_2_ supply.

In order to reveal the detailed therapeutic mechanism through tumor‐pathological analysis, H&E, DCFH‐DA, and Terminal Deoxynucleotidyl Transferase‐mediated fluorescein‐dUTP Nick‐End Labeling (TUNEL) fluorescence staining was used. H&E and TUNEL staining showed that the tumor tissues in the LOD@HFN and Syr/LOD@HFN groups were severely damaged and necrotic (Figure 4I). These results demonstrated that the Syr/LOD@HFN acted as an effective nanozyme to inhibit tumor growth by accelerating catalytic reactions via continuously replenishing raw materials. In addition, DCFH‐DA staining results directly displayed that the Syr/LOD@HFN produced the highest ROS level. It proved that the increasing residual lactate within the tumor could significantly improve the efficiency of catalytic therapy in vivo. More importantly, the approach to enhanced catalytic therapy by lowering intracellular pH was applicable to most nanocatalysts, which greatly increases the practicability of the strategy.^[^ ^19^, ^37^, ^41^ ^]^

### 2.5 In Vitro and In Vivo Anti‐Tumor Immune Microenvironment Remodeling by the Syr/LOD@HFN Nanozyme Platform

Lactic acid is a major contributor to the formation of the immunosuppressive tumor microenvironment, and functions of NK cells, effector T cells, macrophages, and Treg cells are all directly related to the lactic acid in the extracellular matrix.^[^ ^3^, ^5^, ^6^, ^15^ ^]^ In addition, excessive ROS can cause the ICD of tumor cells and release various inflammatory factors to activate tumor immunity.^[^ ^8^, ^42^ ^]^ In this study, a two‐pronged approach to the complete reversal of the ISTME was applied.

**Figure 5:**
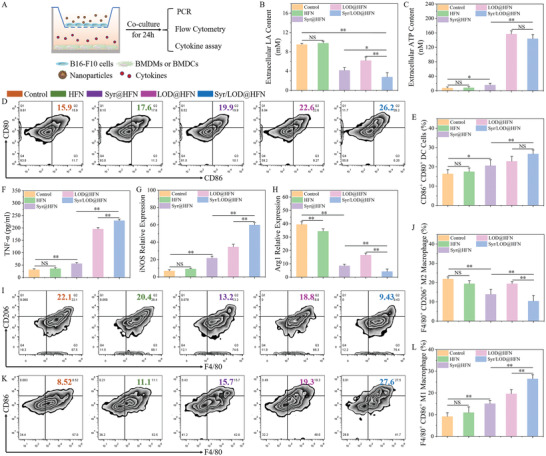
In vitro stimulation of immune response of bone‐marrow‐derived macrophages (BMDMs) or bone marrow‐derived dendritic cells (BMDCs) incubated with the B16‐F10 cells upon the multifarious nanoparticle's treatment. A) Schematic illustration of the transwell system experiment. The B16‐F10 cells and their residues were placed in the upper chamber, and BMDMs or BMDCs were cultured in the lower chamber. Extracellular contents of B) lactic acid and C) ATP in supernatant samples of the B16‐F10/BMDCs co‐culture system after incubation with various nanoparticles (100 µg ml^−1^) for 24 h. D) Flow cytometry analyses and E) quantitative analyses of the representative receptors CD86^+^ and CD80^+^ of mature DCs. F) Quantification of the secretion levels of TNF‐*α* in B16‐F10/BMDMs co‐culture system suspensions. The relative level of G) iNOS mRNA and H) Arg1 mRNA of BMDMs in the B16‐F10/BMDMs co‐culture system. I) Flow cytometry analyses and J) quantitative analyses of the representative receptors F4/80^+^ and CD206^+^ of M2 macrophages. K) Flow cytometry analyses and L) quantitative analyses of the representative receptors F4/80^+^ and CD86^+^ of M1 macrophages. (p‐Values were calculated by using unpaired *t*‐test, *n* = 3, ^*^ *p* < 0.05, ^**^ *p* < 0.01).

First, an experiment based on a typical transwell co‐culture system verified the improvement of the nanosystem on the tumor immunosuppressive microenvironment in vitro, focusing on macrophage polarization and dendritic cell maturation.^[^ ^43^ ^]^ As shown in **Figure** **5**A, the B16‐F10 cells were incubated with respective bone‐marrow‐derived macrophages (BMDMs) and bone‐marrow‐derived dendritic cells (BMDCs) in a transwell co‐culture system incorporating various types of nanoparticles, after which immune responses of the macrophages and dendritic cells were assessed. In the co‐culture system, lactate secretions by the Syr@HFN and Syr/LOD@HFN treated cells were significantly reduced, especially in the Syr/LOD@HFN group with a combination of the inhibition of lactate efflux and lactate consumption (Figure 5B). Subsequently, we examined the concentration of ATP released from the tumor cells, and the results showed that the cells treated by respective LOD@HFN and Syr/LOD@HFN released a large amount of ATP from the damaged cells due to their ability to produce plenty of ROS (Figure 5C). ATP, as one of the important damage‐associated molecular pattern molecules, participates in activating immunity.^[^ ^44^ ^]^ Moreover, flow cytometry was utilized to confirm the high calreticulin (CRT) expression induced by LOD@HFN and Syr/LOD@HFN (Figure S19, Supporting Information). As a dominant biomarker during ICD, CRT acts as an “eat me” signal to guide phagocytes to take up the dying tumor cells.^[^ ^44^ ^]^

In addition, the immunogenicity of tumor cells on the maturation of the DCs with the various treatments was assessed in vitro. The maturation of DCs was verified by the costimulatory molecules CD80 and CD86, which were considered markers for eliciting T cell‐mediated immune responses via antigen presentation.^[^ ^45^ ^]^ The DCs maturation frequency (CD11c^+^CD80^+^CD86^+^) of the control group was 16.4%, which sharply increased to 26.7% in the Syr/LOD@HFN‐treated BMDCs group (Figure 5D,E).

Furthermore, macrophage M1 polarization exhibits positive anti‐tumor effects upon directly phagocytosed tumor cells and secretion of a variety of immunomodulatory cytokines, especially tumor necrosis factor ‐*α* (TNF‐*α*), which enables direct inhibition of the tumor cells without significant toxicity to the normal cells.^[^ ^43^ ^]^ Therefore, concentrations of the TNF‐*α* secreted by the B16‐F10/BMDMs co‐culture system under multiple treatments were first examined. ELISA results showed the highest level of TNF‐*α* in the supernatant of the Syr/LOD@HFN treated cells (Figure 5F). Polymerase chain reaction (PCR) results showed that expression of the inducible nitric oxide synthase (iNOS) was significantly upregulated in BMDMs from the Syr/LOD@HFN‐treated B16‐F10/BMDMs co‐culture system, which controls the release of the cytotoxic NO in the macrophages (Figure 5G). Moreover, BMDMs in the Syr/LOD@HFN group showed a dramatic decrease in the Arg1 expression responsible for inhibiting the cytotoxic NO release (Figure 5H).^[^ ^43^ ^]^ These results indicated that BMDMs co‐cultured with the Syr/LOD@HFN‐treated B16‐F10 substantially enhance the antitumor activity.

Subsequently, the polarization of the macrophages was investigated by flow analysis. M1 macrophages were identified by F4/80^+^CD86^+^ signal, while M2 macrophages were by F4/80^+^CD206^+^ signal. These results showed that the Syr/LOD@HFN group exhibited not only the lowest percentage of M2 type macrophages (Figure 5I,J) but also an increase of the percentage of M1 type macrophages by about twofold compared with the control group (Figure 5K,L). Moreover, detailed analysis revealed that the lactate in the tumor microenvironment directly regulated macrophage M2 polarization. Thus, it is understandable that the Syr@HFN group induced a lower proportion of M2‐type macrophages than the LOD@HFN group. Whereas M1 polarization of the macrophage depended more on the ROS content, thus the proportion of the M1 macrophages in the LOD@HFN group was lower than that in the Syr/LOD@HFN group.^[^ ^46^ ^]^

Taken together, in the aforementioned serial in vitro experiments, the Syr/LOD@HFN was proven to be the most efficient group in stimulating DC cell maturation and promoting macrophage M1 polarization due to its combination of inhibition of lactate efflux, and stimulation of release of inflammatory factors release as well.

To investigate the feasibility of this strategy of sequestering lactate inside tumor cells for remodeling the ISTME in vivo, the efficacy of the Syr/LOD@HFN on tumor immunoreactivity reactivation was examined using the B16‐F10 C57/BL6 mice tumor model. In vivo imaging data proved that nanocarriers can accumulate at the tumor site even in immunocompetent C57/BL6 mice, where fluorescence intensity is strongest at 12 h (Figure S20, Supporting Information). By semi‐quantitative analyses of fluorescence intensities of serial mice blood samples collected at different time intervals after systemic administrations of the Cy5.5‐labeled Syr/LOD@HFN, a classical two‐compartment model of the blood circulation profiles of the nanoplatform is observed (Figure S20D, Supporting Information).^[^ ^47^ ^]^ The half‐lives of the Syr/LOD@HFN were 1.41 h by the in vivo pharmacokinetic assay. Good circulatory function in vivo and efficient enrichment in the tumor site were prerequisites for the reversal of ISTME.

Lactic acid contents in both serum and tumor interstitium were detected, and the results showed that lactic acid concentration was significantly reduced in both serum and tumor interstitium after treatments (Figures S21 and S22, Supporting Information). Moreover, the pH of the tumor microenvironment was increased from 6.43 to close to neutral 6.82 (Figure S22, Supporting Information). Furthermore, the tumor interstitial pH value was negatively correlated with the lactate content in the tumor stroma, indicating that the change in pH was directly related to the inhibition of lactate efflux by the nanosystem.

**Figure 6:**
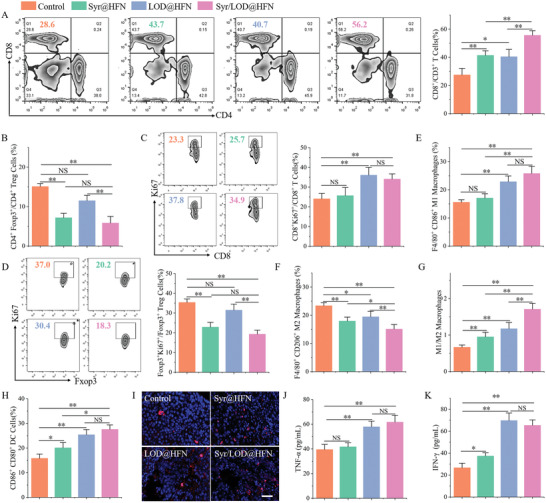
Evaluation of tumor immune activation in vivo after various treatments. Representative flow cytometric zebra plots and corresponding quantification of intratumoral populations of A) CD8^+^ T cells (gated on CD3^+^ T cells), B) Foxp3^+^ Treg cells, C) Ki67^+^ CD8^+^ cells, and D) Ki67^+^ Treg cells of B16‐F10 tumor‐bearing mice with different treatments as indicated and analyzed (*n* = 4). The ratio of E) CD86^+^ M1 macrophages and F) CD206^+^ M2 macrophages versus F4/80^+^macrophages. G) The ratio of M1 to M2 macrophages in different treatment groups. H) The proportion of mature DC cells in lymph nodes adjacent to the tumor. I) Representative CLSM images of the tumors under various treatments labeled by NK cells immunofluorescence staining. Scale bar: 50 µm. Quantification of the J) TNF‐*α* and K) IL‐1*γ* levels in serum by ELISA after the designated treatments. Data are expressed as mean ± SD (*n* = 4). Data are present as mean ± SEM. (p‐Values were calculated by using unpaired *t*‐test, *n* = 4, ^*^ *p* < 0.05, ^**^ *p* < 0.01).

Lower lactate levels in the tumor microenvironment were considered a positive sign for immune activation. Subsequently, immune responses were evaluated, including typical cellular immunity (T cells) and innate immunity (macrophages) in the tumor with different treatments. As partially shown in **Figure** **6**A, the average percentage of the cytotoxic T cells in the tumor tissue treated by the Syr/LOD@HFN was 55.7%, while the counterpart value in the control group and the Syr@HFN group were 27.6% and 41.5%, respectively. Moreover, the percentage of Treg cells in the group of the Syr@HFN and Syr/LOD@HFN decreased by 53% and 58%, respectively, compared to the control group (Figure 6B; Figure S23, Supporting Information), which proposed strongly positive implications for activating the immunity and suppressing tumor metastasis.^[^ ^22^, ^48^ ^]^ More importantly, the cytotoxic T cell activity was also enhanced in the Syr/LOD@HFN group, with a 48% increase in the proportion of Ki67 positivity (Figure 6C). Accordingly, the proportion of Treg cells Ki67 positivity in such group decreased by 45% (Figure 6D). These led to the reactivation of anti‐tumor immune activity. The realization of synergistic promotion of the anti‐tumor immune activities from the opposite directions involving both Treg cells and cytotoxic T cells was due to the different tolerance of the two types of cells to lactate and the adaptability of Treg cells to take up and utilize lactate.^[^ ^3^ ^]^

Moreover, tumor‐associated macrophage (TAM) phenotypes were characterized to examine their anti‐tumor activity. The results of flow cytometric analysis showed that the percentage of M2 macrophages significantly decreased in all treatment groups, while the percentage of M1 macrophages was significantly different between LOD@HFN, Syr/LOD@HFN, and the control group (Figure 6E,F; Figures S24 and S25, Supporting Information). The final manifestation was that the ratio of M1/M2 macrophages in the Syr/LOD@HFN group was significantly higher than that in the other three groups (Figure 6G), which benefits enhanced tumor inhibition.^[^ ^4^, ^14^ ^]^

The ability of nanoplatform to activate immune responses was further validated by detecting DC maturation. As presented in Figure 6H and Figure S26 (Supporting Information), compared with the control group, a slight increase of the DC maturation (CD11c^+^/CD80^+^/CD86^+^) in the Syr@HFN group (20.1% to 15.8%) was discovered. The percentage of the mature DCs in the LOD@HFN group amounted to 25.4% due to the promotion by the stimulation of ROS induced by a bunch of inflammatory factors.^[^ ^49^ ^]^ Moreover, the number of mature DCs increased in the Syr/LOD@HFN treatment group to 27.6%, suggesting that the ICD was the major factor inducing the DC maturation (Figure 6J,K).

Immunofluorescence staining results showed that natural killer (NK) cells (NK1.1^+^) in the group of Syr/LOD@HFN were more infiltrated into the tumor (Figure 6I; Figure S27, Supporting Information), which may be due to lower lactate tolerance of NK cells and reduced activity at high concentrations of lactate.^[^ ^6^ ^]^ As the first line of defense against cancer, NK cells can target and kill tumor cells in the early stage, with great significance for the prevention and treatment of tumors.^[^ ^50^ ^]^

Taken together with the Syr/LOD@HFN nanozyme platform, the integrated anti‐tumor immune activities were greatly reactivated, including reactivation of effector T cells and NK cells, inhibition of Treg cells, promotion of M1 polarization of macrophages, and DC maturation. The reason for the remarkable effects was that delivered Syr significantly reduced the concentration of lactic acid in both serum and tumor stroma (Figures S21 and S22, Supporting Information), accompanied by an increase in the concentration of cell proinflammatory cytokines (TNF‐*α* and IFN­*γ*) in serum with the help of the LOD induced ICD of tumor cells (Figure 6J,K).

### 2.6 The Synergetic Anti‐Tumor Nanocatalytic Activity and Augmented Immune Checkpoint Blockade Induced by the Syr/LOD@HFN Nanozyme Platform

**Figure 7:**
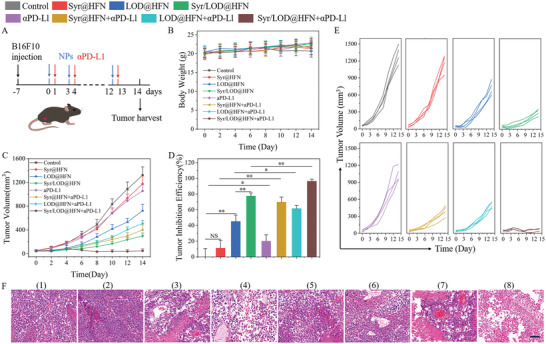
In vivo tumor immune checkpoint blockade therapy. A) Schematic diagram of the B16‐F10 tumor‐bearing mice model and nanoplatform treatments. B) Average body weights, C) tumor growth curves, and D) tumor inhibition efficiency of the B16‐F10 tumor‐bearing mice with various treatments. E) Corresponding individual growth curves for the indicated treatments. F) H&E staining for pathological changes in tumor tissues from each group after a 14‐day therapeutic period. Scale bar: 100 µm. Data are present as mean ± SEM. (p‐Values were calculated by using unpaired *t*‐test, *n* = 4, ^*^ *p* < 0.05, ^**^ *p* < 0.01).

Tumor immunotherapy, as one of the most promising strategies to cure tumors, is only partially effective in solid tumors due to the immunosuppressive microenvironment in solid tumors.^[^ ^51^ ^]^ The ISTME had been fundamentally reversed by the nanoplatform, which may have important positive significance for enhancing the therapeutic effect of immune checkpoint blockade (ICB) therapy. To this end, the combined anti‐tumor efficiency of the Syr/LOD@HFN platform with programmed cell death protein ligand 1 blockade immunotherapy (*α*‐PD‐L1) was further investigated. The B16‐F10 tumor­bearing mice were randomly divided into eight groups, and tumor treatment was started from day 7 after tumor inoculation and recorded as day 0. Nanocarriers with varied drug loads were injected on days 0, 3, 6, 9, and 12, respectively, and intravenous *α*‐PD‐L1 was administered one day after nanocarriers treatment (**Figure** **7**A). There was no significant change in the body weight of the mice during the whole treatment process, demonstrating the biocompatibility of the nanocarriers again (Figure 7B). Upon *α*‐PD‐L1 monotherapy, the tumor suppression efficiency was mere 20.1% compared with the control group (Figure 7C,D) due to the difficulty of immune cell infiltration in solid tumors and limited anti‐tumor ability.^[^ ^52^ ^]^ However, when the Syr@HFN and *α*‐PD‐L1 were used together, the tumor inhibition rate increased sharply to 69.7% (CDI = 0.42). Contrarily, the tumor inhibition rate only reached 61.5% for the combination of the LOD@HFN and *α*‐PD‐L1, which was slightly higher than that of 45.1% for the LOD@HFN treatment group (Figure 7E), and the CDI coefficient was 0.88, indicating a low degree of synergy. In addition, the Syr/LOD@HFN plus *α*‐PD‐L1 group achieved the highest tumor inhibition rate (96.3%) and the lowest CDI coefficient (0.21), indicating the highest synergistic effect. These results suggested that the inhibition of lactate efflux by Syr has a critical role in improving the therapeutic effect of immune checkpoint blockade (ICB) by remodeling the ISTME.

To further evaluate the therapeutic effect, the tumor tissues of the different groups were collected for the H&E staining. As shown in Figure 7F, the largest damaged area was found in the tumor tissue of the Syr/LOD@HFN plus *α*­PD‐L1 group, implying the good anti‐tumor effect of the combination therapy.

Intriguingly, the strategy of the Syr/LOD@HFN nanoplatform to envelop lactic acid inside tumor cells and the tumor ICD driven by augmented catalytic therapy effectively activated anti‐tumor immune activity, thus overcoming the limitation of ICB therapy in solid tumor treatment to some extent.

## 3 Conclusion

In summary, we reported a novel one‐stone‐two‐birds strategy to construct a dual‐drug co‐delivery nanozyme platform Syr/LOD@HFN with a synergy of self‐replenishing enhanced nanocatalytic reactivity, integrate starvation therapy, and exertion of immunosuppressive tumor microenvironment remodeling via highly efficient inhibition of the lactate efflux by the loaded drug Syr as the trigger. Cascade utilization of the increased residual lactic acid by the Syr/LOD@HFN enabled the synergy of accelerated nanocatalytic reaction and sustainable raw material supply, integrated starvation tumor therapy, and dramatically amplified ICB therapy. The multifunctional Syr/LOD@HFN nanoplatform was prepared in a reproducible and facile way and exhibited good biocompatibility, through which the coordinated multi‐bio‐effects inside the tumor cells and TME were achieved.

Statistical analysis was done using a two‐tailed, unpaired Student's *t*‐test (two groups) or one‐way ANOVA (three or more groups) with SSPS Statistics 26 software. Statistical significance was annotated with ^*^ *p* ≤ 0.05 and ^**^ *p* ≤ 0.01, respectively. Quantitative data are represented as mean ± SEM.

## Conflict of Interest

The authors declare no conflict of interest.

## Supporting information

Supporting Information

## Data Availability

The data that support the findings of this study are available from the corresponding author upon reasonable request.
