# Supplementary material for: Lactate Efflux Inhibition by Syrosingopine/LOD Co‐Loaded Nanozyme for Synergetic Self‐Replenishing Catalytic Cancer Therapy and Immune Microenvironment Remodeling
Source: Adv Sci (Weinh). 2023 Jun 29;10(26):2300686. doi: 10.1002/advs.202300686 (PMC10502866; doi:10.1002/advs.202300686)
Supplement: Supplementary file 1 — Supporting Information [file ADVS-10-2300686-s001.pdf]

ADVANCED SCIENCE
Open Access

Supporting Information

for *Adv. Sci.*, DOI 10.1002/advs.202300686

Lactate Efflux Inhibition by Syrosingopine/LOD Co-Loaded Nanozyme for Synergetic Self-Replenishing Catalytic Cancer Therapy and Immune Microenvironment Remodeling

*Shengming Wu, Lehua Xu, Chenlong He, Peng Wang, Jingwen Qin, Fangfang Guo and Yilong Wang**

Supporting Information

Lactate efflux inhibition by syrosingopine/LOD co-loaded nanozyme for synergetic self-replenishing catalytic cancer therapy and immune microenvironment remodeling

*Shengming Wu, Lehua Xu, Chenlong He, Peng Wang, Jingwen Qin, Fangfang Guo, Yilong Wang**

The Institute for Translational Nanomedicine, Shanghai East Hospital, The Institute for Biomedical Engineering and Nano Science, School of Medicine, Tongji University, Shanghai 200092, P. R. China.

E-mail: yilongwang@tongji.edu.cn

Experimental Section

Materials: Acetone, ethyl alcohol and Sodium Carbonate (Na_2CO_3) were purchased from Shanghai (China) Reagent Company. DCFH-DA, polyacrylic acid (PAA, MW≈1800), 3,3′,5,5′-Tetramethylbenzidine (TMB), fluorescein isothiocyanate (FITC), ferrocene and (3-Aminopropyl) triethoxysilane (APTES) were purchased from Sigma-Aldrich (USA). Tetraethyl orthosilicate (TEOS), Metformin, Cy5.5 NHS ester and poly(allylamine hydrochloride) (PAH, MW≈15,000), were purchased from Aladdin Reagent Co, Ltd. Rhodamine-phalloidin and 4',6-diamidino-2-phenylindole (DAPI) were purchased from Shanghai Yeasen Biotech Co, Ltd. RPMI-1640 medium, penicillin-streptomycin and 0.25% trypsin-EDTA were purchased from Gibco Corp. ATP Assay Kit, RIPA Lysis Buffer, Hydrogen Peroxide Assay Kit, Lyso-Tracker Green, NAD+/NADH Test Kit (WST-8 Method), MCT1 Rabbit Polyclonal Antibody, Mitochondrial Membrane Potential Assay Kit with TMRE, Annexin V-FITC Apoptosis Detection Kit and BCECF AM were purchased from Beyotime Biotechnology Corp. Fetal bovine serum (FBS) were purchased from MRC Corp. Dulbecco's Modified Eagle's medium (DMEM) and phosphate-buffered saline (PBS) were purchased from Hyclone Corp. PEG polymers were obtained from JiaXing BoMei, China. Lactate oxidase (LOD), AZD3965, VB124 and Syrosingopine (Syr) were obtained from Shanghai Yuanye Bio-Technology Co., Ltd. Lactic Acid assay kit was obtained from Nanjing Jiancheng Bioengineering Institute. Anti-Calreticulin were purchased from Abcam Corp. APC-anti-mouse CD3, PE-anti-mouse CD8a, FITC-anti-mouse CD4, PE-anti-mouse FOXP3, APC-anti-mouse F4/80, FITC-anti-mouse CD86, FITC-anti-mouse CD206, APC-anti-mouse CD11c, PE-anti-mouse CD80 and True-Nuclear Transcription Factor Buffer Set were purchased from Biolegend Corp.

Characterization: JEM-2010 transmission electron microscopy (TEM) was used to characterize the morphology of nanoparticle. Dynamic light scattering (DLS) measurements were carried out at 298.0 K using Zetasizer Nano-ZS (Malvern, UK) equipped with standard 633 nm laser. X-ray diffraction (XRD) patterns of materials were obtained on a Rigaku Ultimate IV X-ray diffractometer over the 2q range 10-80°.

Synthesis of PEGylated hollow Fe_3O_4 nanoparticles (HFN): (1) SiO_2 nanoparticles were prepared according to the Stöber method.[1] (2) The SiO_2 nanoparticles serve as a template to construct the hollow nanostructures.[2] Briefly, 75 mg of SiO_2 nanospheres and 300 mg of ferrocene were dispersed in 90 mL of acetone and the mixed solution was sonicated and stirring for 20 min. H_2O_2 (3.0 mL, 30%) was then added into the solution dropwise under sonication with being stirred for 2 h. After that, the mixture was transferred into a 150 mL Teflon-lined stainless autoclave. The temperature of the autoclave was maintained at 200℃ for 48 h. The resultant SiO_2@Fe_3O_4 nanoparticles were collected by centrifugation and washed three times with ethanol and water. (3) The as-prepared SiO_2@Fe_3O_4 was dissolved in 2 M Na_2CO_3 aqueous solution at 60℃ for 6 h. The hollow Fe_3O_4 nanoparticles (HMNP) were obtained by magnetic separation and washed three times with deionized (DI) water. (4) For PEG modification, 10 mL HMNP solution (2 mg/mL) was then added to 20 mL PAH solution (5 mg/mL) under ultrasonication. After 2 h of stirring, the above solution was centrifuged and washed with deionized water, and subsequently the obtained nanoparticles were added to 20 ml of PAA solution (5 mg/mL) for another 2 h reaction. The

PAA-modified nanoparticles were obtained and mixed with 50 mg mPEG-5K-NH_2 under sonication for 30 min. After adding 15 mg EDC and stirring for 12 h, the prepared hollow Fe_3O_4-PEG (named HFN) was collected by centrifugation and washed with deionized water for three times.

Synthesis of Cy5.5 conjugated nanoparticles: For the Cy5.5-labeled nanoparticles, it was only necessary to mix 1mg HMNP-PAH with 50 μg Cy5.5-NHS for 12h. After the nanoparticles were collected by centrifugation, PAA and PEG modification were performed according to the same procedure "*Synthesis of PEGylated hollow Fe_3O_4 nanoparticles* (4)".

Synthesis of Syr/LOD@HFN: For Syr loading optimization, a variety of volumes of Syr DMSO solution (10 mg/mL) ranged from 2.5 to 25 µL were used based on 1 mg of HFN. In the typical process of preparation of Syr/LOD@HFN and Syr@HFN, 20 µL of the Syr DMSO solution was mixed with HFN for 1 h. Finally, the Syr@HFN were obtained by centrifugation and washed with DI water three times. For LOD loading, 1 mg of Syr@HFN or HFN were dispersed in 1 mL of DIW, 50 μg of LOD was added and the reaction was overturned overnight. The nanoparticles were subsequently washed by centrifugation three times to obtain the Syr/LOD@HFN or LOD@HFN. The supernatant of each wash was collected to determine the eluted LOD or Syr concentration and the drug loading rate was determined by the differential subtraction method.

LOD and Syr release detection: 1 mg of nanoparticles were dispersed in 10 ml of PBS with different pH values (5.5, 6.5, 7.4) and 1 ml of the solution was collected at the time 10 min, 30 min, 1 h, 2 h, 4h, 12h, 24h after centrifugation to remove the nanoparticles, and the Syr content was determined by detecting UV absorption (257 nm) and the LOD content was determined by using the BCA kit.

Cell Culture: B16-F10 mouse melanoma cells were obtained from the Type Culture Collection Committee of the Chinese Academy of Sciences (Shanghai, China), and was cultured at 37 °C (5% CO_2) in DMEM supplemented with 10% v/v FBS and 1%PS.

Intracellular pH measurement: Intracellular pH was determined with BCECF-AM. B16-F10 cells grown in DMEM containing 10% FBS were seeded in 24-well plates (50000 cells/well) for 12 h. After NPs (HFN 100 μg/ml; LOD 1.05 μg/ml; Syr 16.1 μg/ml) treatment for 6 h, cells were washed with PBS and labeled with BCECF-AM for 30 min. After washing with PBS, fluorescent images were then taken using an inverted fluorescence microscope (488/535 Ex/Em) and intracellular pH determined according to calibration standards (Thermo Fisher Scientific, P35379).

Western Blot Analysis on the Changes in MCT1 and MCT4 Levels: B16-F10 cells were seeded in a 6-well plate with a density of $2x10^5$ and incubated overnight. The cells were then treated by variety of nanoparticles at the concentration of 100 μg/mL for 24 h. The total protein concentration was determined by BCA protein assay kit. Protein immunoassay was performed by SDS-PAGE electrophoresis and finally photographed by a molecular imaging apparatus to determine the expression levels of MCT1and MCT4.

Intracellular ATP and NAD^+/NADH detection: B16-F10 cells were seeded into 24-well plates at a density of 50000 cells per well and cultured overnight. Then the culture media were replaced by fresh medium containing NPs (50 µg/mL) incubation for 24 h. Cells were lysed using Ripa lysis solution, and ATP content was measured by the enhanced ATP Assay Kit according to the instructions and simultaneous measurement of protein content. The intracellular content of NAD^+ and NDAH was detected using the NAD^+/NADH Test Kit (WST-8 Method), and the experimental procedure was in full accordance with the instructions protocol.

Cytotoxicity of nanosystem: B16-F10 cells were seeded into 96-well plates at a density of 10000 cells per well and cultured overnight. Then the culture media were replaced by fresh medium containing Syr or not incubation for 6 h. The medium was then replaced with glucose-free DMEM medium containing different nanoparticles (100 µg/mL). After further incubation for 24 h, the culture media were replaced by FBS-free medium containing 10% CCK-8. After further co-incubation for 1 h, the cell proliferation was determined using the microplate reader by comparing the absorbance at 450 nm to the control group.

Intracellular Endocytosis Analysis: For CLSM observation, B16-F10 cells were seeded (1×10^5 cells in 1 mL DMEM) in the culture dishes and allowed to adhere overnight. Then the culture media were replaced by Cy5.5-labeled NPs (1 mL, 50 µg/mL) in DMEM. After varied co-incubation durations (0, 1, 2 and 4 h), the cells were labeled with DAPI.

Characterization of co-localization of nanoparticles with lysosomes: B16-F10 cells were seeded (1×10^5 cells in 1 mL DMEM) in the culture dishes and allowed to adhere overnight. Then the culture media were replaced by Cy5.5-labeled NPs (1 mL, 50 µg/mL) in DMEM. After 4 h of incubation, DAPI was used to label nuclei and Lyso-Tracker Green to label lysosomes. Cells were photographed using confocal microscopy immediately thereafter.

Intracellular ROS Evaluation: Inverted fluorescence microscope was introduced to evaluate the ROS generation. B16-F10 cells were seeded (1×10^5 cells in 1 mL DMEM, pH 6.5) in the 24-well cell culture plate and allowed to adhere overnight. The culture media were then replaced by 1 mL of acidulated DMEM as the following groups: 1) control; 2) 50 µg/ml HFN; 3) 50 µg/ml Syr@HFN; 4) 50 µg/ml LOD@HFN; 5) 50 µg/ml Syr/LOD@HFN. After incubation for 2 h, the culture media were replaced by 1 mL DCFH-DA (10 µM) and incubated for 30 min. The cells were washed with PBS three times and the intracellular ROS was evaluated with Inverted fluorescence microscope.

Detection of intracellular and extracellular lactic acid content: Lactic Acid assay kit was adopted to quantify the lactic acid content. Typically, B16-F10 cells (1×10^6) were cultured in 6-well plates. After treatments with various NPs (50 µg/mL, pH 6.5) for 6 h or 24 h, the cells were collected by trypsinization and washed twice with PBS and followed ultrasonic cell fragmentation. Then, the supernatant was collected by centrifugation and tested according to the provided protocol. Finally, the relative lactic acid content was analyzed using a microplate reader at the absorbance of 530 nm. Extracellular lactate concentration was obtained by direct assay of the culture medium. For the effect of Syr@HFN on lactate efflux at different pH (pH 7.4 or pH 6.5), the cells were collected

for lactate content assay immediately after 6 h of incubation with nanoparticles.

Animal models: Female Balb/c Nude mice and C57/BL6 mice (5-6 weeks) were purchased from Shanghai Laboratory Animal Center (SLAC, Shanghai, China) and bred in a sterilized, specific pathogen-free (SPF) Lab of Tongji University. All animal procedures conformed to the Guide for the Care and Use of Laboratory Animals.

Evaluation of anti-tumor efficacy of catalytic therapy in vivo: The 6-week-old female Balb/c Nude mice (~ 20 g) were obtained and raised at Laboratory Animal Center, Tongji University school of Medicine. To develop the animal tumor model, xenografted tumors were generated in 6-week-old female Balb/c mice by subcutaneously (s.c.) injecting of B16-F10 cells (1×10^6 cell/site, 100 μL DMEM medium) into the right rear legs of mice. Once the tumor volume reached nearly 50 mm^3, the tumor-bearing mice were randomly divided into five groups (n = 4): 1) Control; 2) Free Syr; 3) Syr@HFN; 4) LOD@HFN; 5) Syr/LOD@HFN, (intravenous injection, dose 20 mg/kg). NPs were injected on day 0, day 3, day 6, day 9 and day 12. The length and width of tumors were measured by digital caliper every two days during half a month post injection. The tumor volume was calculated according to the following formula: tumor volume = $L \times W^2/2$, where L and W mean the length (mm) and width (mm) of tumor, respectively. The tumors were dissected and sliced for further hematoxylin and eosin (H&E) staining assay for observing the structure and status of cells, terminal deoxynucleotidyl transferase mediated dUTP nick-end labeling (TUNEL) staining assay for detecting DNA fragmentation and Ki-67 antibody staining assay for detecting the growth fraction of cells.

The B16-F10 tumor-bearing mice were randomly divided into five groups (n = 4): 1) Control; 2) Free Syr; 3) Syr@HFN; 4) LOD@HFN; 5) Syr/LOD@HFN, (intravenous injection, dose 20 mg/kg). NPs were injected on day 0, day 3, day 6, day 9 and day 12. The length and width of tumors were measured by digital caliper every two days during half a month post injection. The tumors were dissected and sliced for further H&E staining assay for observing the structure and status of cells, TUNEL staining assay for detecting DNA fragmentation and DCFH-DA staining assay for detecting the ROS of tumors.

Tumor lactate and ATP and content level assessment in vivo for B16-F10 tumor-bearing nude mice model: In nude mice after tumor growth to 100 mm^3, tumors were dissected in 24 h post-injection (intravenous injection, dose 20 mg/kg). Tumors were weighed and then cut up and lysed using Ripa lysis solution, and finally lactate content was determined using lactate assay kit and ATP content was determined using ATP Assay Kit.

Combination index calculation: The combination index (CI) was calculated according to a reported formula[6]

$$CI = \frac{AB}{A \times B}$$

A or B was the tumor survival rate under single therapy (e.g. Syr@HFN or LOD@HFN), and AB was the tumor survival rate under the corresponding combination therapy.

Acquisition of bone-marrow-derived macrophages (BMDMs): BMDMs were extracted from the

femur and tibias of WT mice (6-8 weeks) by flushing sterile PBS to the end of the bone using a syringe. The extract was centrifuged at 2,000 rpm for 5 min and then the pellet was resuspended in RPMI 1640 supplemented with 10% FBS, 1% antibiotics, containing mouse M-CSF (20 ng/ml) to prepare cell suspensions at 1×10^6 cells/ml. Media was changed on day 3 and 5 and Cells were used for experiments at day 6 of culture.

Acquisition of bone-marrow-derived DCs (BMDCs): BMDCs were extracted from the femur and tibias of WT mice (6-8 weeks) by flushing sterile PBS to the end of the bone using a syringe. In brief, BMDC cells (2×10^6 mL^{-1}) were cultured into 6-well plates in 4 ml of RPMI 1640 complete medium supplemented with GM-CSF (20 ng/mL) and IL-4 (10 ng/mL). On day 2 and 4, culture medium was removed and replaced with fresh medium containing GM-CSF and IL-4. At day 6, immature BMDCs were harvested for further experiments.

Co-culture experiment of BMDCs or BMDMs and B16-F10 cells: Transwell plates were used to co-incubate BMDCs or BMDMs with B16-F10 cells under the various treatment of nanocomposites to investigate the immune response of macrophages and DC maturation. Firstly, B16-F10 cells were put in the upper chamber of the transwell system, while the BMDCs or BMDMs were seeded in the lower chamber. After adding the nanoparticles (100 μg/mL) into the upper chamber for 24 h, the supernatant was collected for determination of ATP, lactate and TNF-a concentrations, and immune cells were collected for flow cytometry and PCR.

Tumor stroma and Serum lactate or H_2O_2 assessment in vivo for B16-F10 tumor-bearing mice model: In C57/BL6 mice after tumor growth to 100 mm^3, tumors were dissected in 24 h post-injection (intravenous injection, dose 20 mg/kg). Tumors were collected and weighed, then ground using a 200-mesh filter to obtain a single-cell suspension that was eventually dispersed into 2 mL PBS. The tumor cells were then centrifuged and the supernatant was collected to test lactate concentration and recorded as lactic acid content in the extracellular matrix. At the same time, a fraction of sample was used to directly detect the hydrogen peroxide concentration using the hydrogen peroxide detection kit. For the detection of serum lactic acid, the whole blood was first obtained by taking blood from the heart, and serum was taken after blood coagulation for the detection of lactic acid level.

Hemolysis test: 1 ml of blood was collected from C57/BL6 mice using cardiac blood sampling, and serum was removed by centrifugation three times at 500 g. Subsequently, the cells were dispersed into 50 ml of PBS, and 1 ml was subsequently added to the different nanoparticles. After 1 h of incubation at room temperature, the supernatant was removed by centrifugation at 500 g for 5 min, and the nanoparticles were subsequently removed by centrifugation at 12000 g, and the absorbance at 530 nm was measured using a microplate reader.

pH detection of tumor microenvironment: C57/BL6 mice were inoculated with 2 million B16-F10 cells, and after tumor growth to 500 mm^3, tumors were dissected in 24 h post-injection (intravenous injection, dose 20 mg/kg). The collected tumors were placed in 2 ml physiological saline and ground using a 200 mesh filter to prepare corresponding single cells suspensions. Subsequently, the supernatant was collected by centrifugation at 500 g for 5 min. The pH of

supernatant was measured by an electronic pH meter and used as the pH of tumor microenvironment.

In Vivo Pharmacokinetic Study. To evaluate NPs blood circulation profiles, Cy5.5-labeled Syr/LOD@HFN were i.v. injected into healthy mice (n = 4). At each time point, 15 μL of blood was extracted from each mouse, dissolved in lysis buffer, and then subjected to a microplate reader to measure the Cy5.5 fluorescence.

Evaluation of the effectiveness of immune microenvironment improvement in vivo: In order to comprehensively evaluate the immune microenvironment, immunologically sound C57/BL6 mice were used in this section. The 6-week-old female C57/BL6 mice were obtained and raised at Laboratory Animal Center, Tongji University school of Medicine. To develop the animal tumor model, xenografted tumors were generated in 6-week-old female Balb/c mice by subcutaneously (s.c.) injecting of B16-F10 cells (2×10^6 cell/site, 100 μL DMEM medium) into the right rear legs of mice. Once the tumor volume reached nearly 50 mm^3, the tumor-bearing mice were randomly divided into four groups (n = 4): 1) Control; 2) Syr@HFN; 3) LOD@HFN; 4) Syr/LOD@HFN, (intravenous injection, dose 10 mg/kg). NPs were injected on day 0, day 3, day 6, day 9 and day 12. For flow cytometric analysis, these collected tumors were ground using a 200 mesh filter to prepare corresponding single cells suspensions. After being stained with corresponding fluorescence antibodies by according to the standard protocols, the mature DCs ($CD11c^+CD80^+CD86^+$) inside the lymph nodes, and $CD8^+$ T cells ($CD3^+CD4^+CD8^+$), Tregs ($CD4^+Foxp3^+$), M1 macrophages ($F4/80^+CD80^+$), M2 macrophages ($F4/80^+CD206^+$), proliferating Tregs ($CD4^+Foxp3^+Ki67^+$) and proliferating $CD8^+$ T cells ($CD3^+CD8^+$ $Ki67^+$) inside the tumors were subjected to a flow cytometry for detailed analysis. For the detection of tumor-infiltrating natural killer cells, tumors were dissected and sectioned for NK1.1 staining to label NK cells and DAPI to label nucleus. For cytokines detection, the secretion levels of TNF-α, and IFN-γ in the blood sera were measured by using ELISA kits by according to the manufacturer's instructions.[5]

Evaluation of the effect of nanoplatform-enhanced α-PDL1 immunotherapy: B16F10 tumor-bearing C57/BL6 mice were randomly divided into the following 8 groups, : 1) Control; 2) Syr@HFN; 3) LOD@HFN; 4) Syr/LOD@HFN; 5) α-PDL1; 6) Syr@HFN+α-PDL1; 7) LOD@HFN+α-PDL1 and 8) Syr/LOD@HFN+α-PDL1 (n=4). NPs were injected on day 0, day 3, day 6, day 9 and day 12 (intravenous injection, dose 10 mg/kg). Injected α-PDL1 24 h after NPs injection (intravenous injection, dose 1 mg/kg). During the therapy, tumor size and body weight of the mice were recorded every other day.

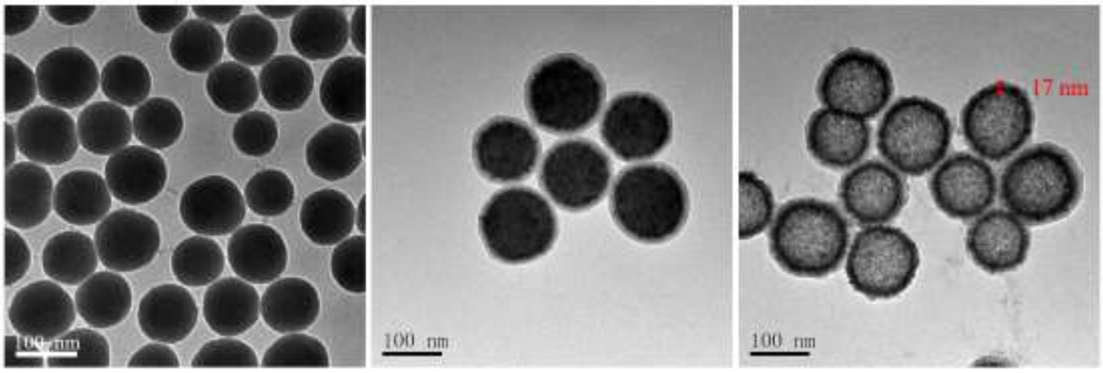

Figure S1. TEM image of (A) SiO_2 nanoparticles, (B) SiO_2@ Fe_3O_4 nanoparticles and (C) Hollow Fe_3O_4 nanoparticles.

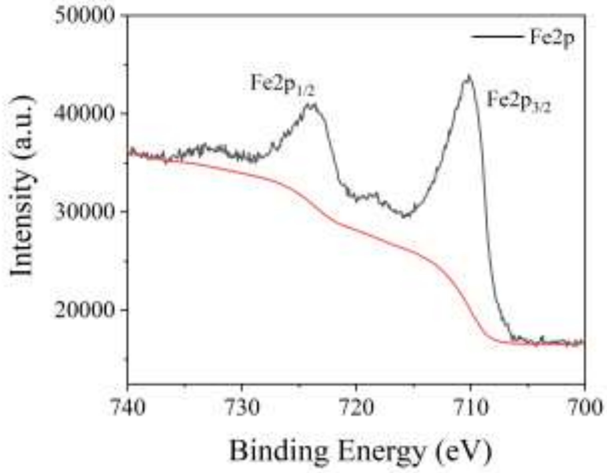

Figure S2. XPS spectra of Fe 2p for HFN.

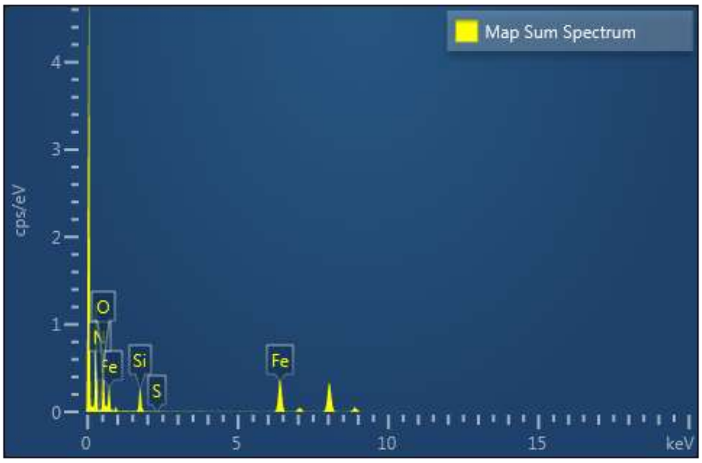

Figure S3. EDS spectrum of Syr/LOD@HFN by STEM.

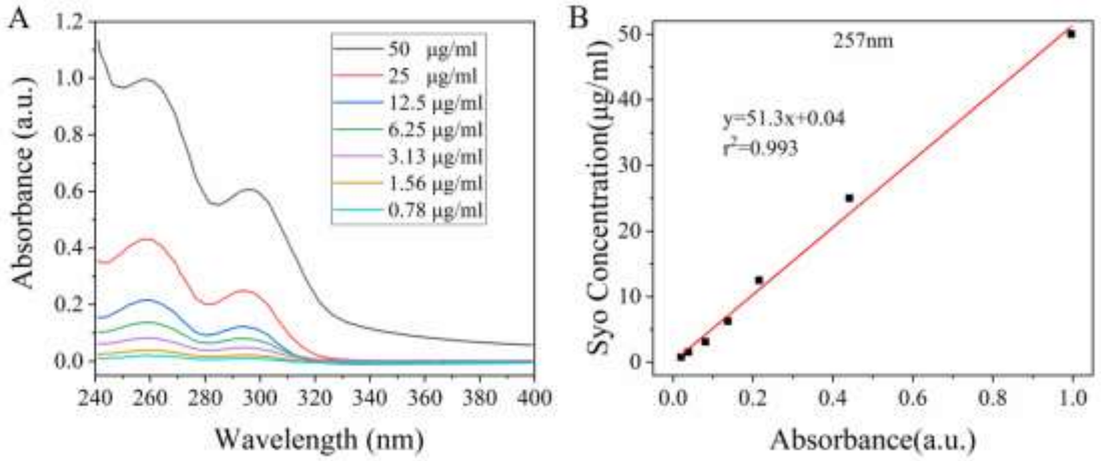

Figure S4. (A) UV-Vis absorption spectra of Syr at different concentrations. (B) The standard curve of Syr linearly fitted between absorption intensity vs Syr concentration.

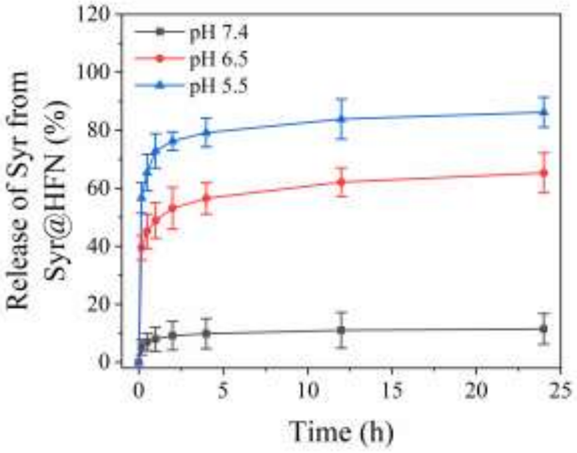

Figure S5. Syr release profiles from the of Syr@HFN in PBS with under different pH conditions.

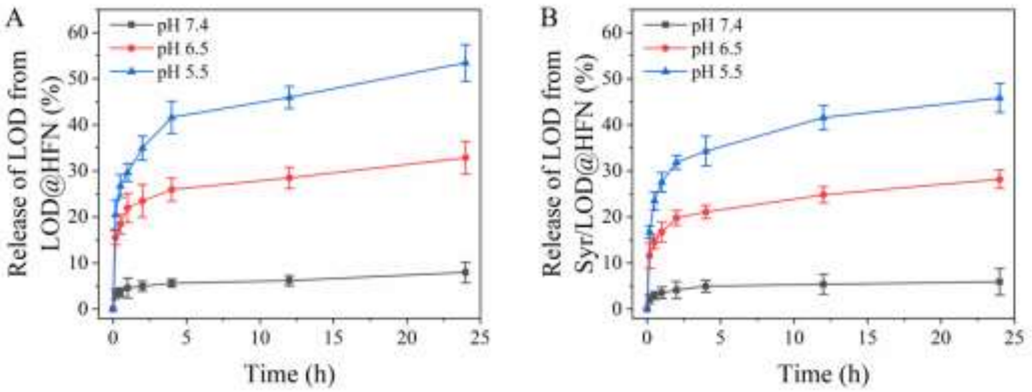

Figure S6. LOD release profiles from the (A) LOD@HFN and (B) Syr/LOD@HFN in PBS under the different pH conditions.

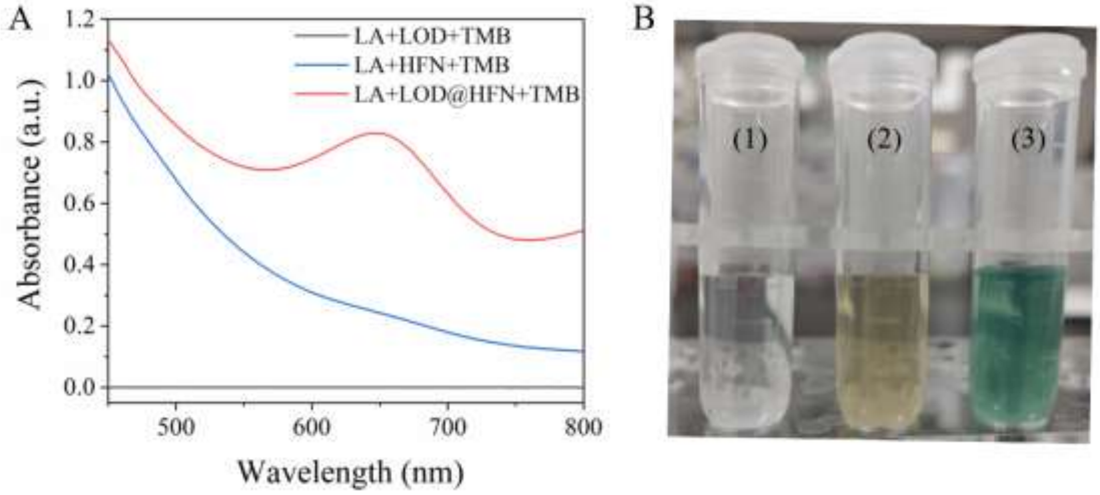

Figure S7. Peroxidase-like activity of the LOD@HFN. (a) Absorbance at 650 nm of TMB reaction solutions catalyzed by the LOD@HFN and LA at room temperature for 30 min (PBS, pH 6.5). (b) Photographs for the chromogenic reaction of TMB catalyzed by the LOD@HFN. (1) LA+LOD+TMB; (2) LA+HFN+TMB; (3) LA+ LOD@HFN +TMB.

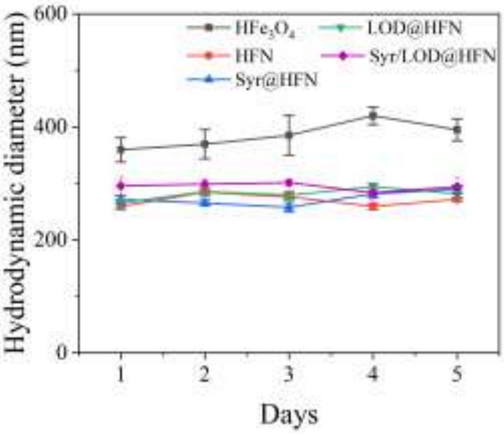

Figure S8. Colloidal stability of the different nanoparticles dispersed in FBS (pH 7.4).

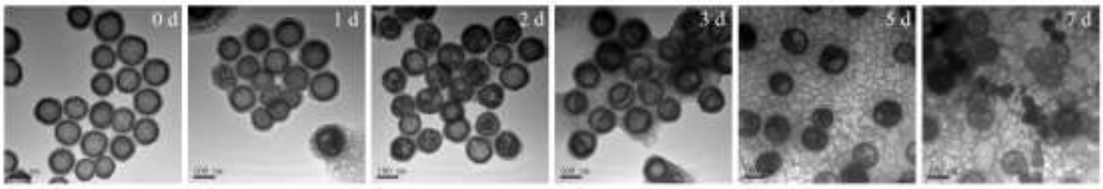

Figure S9. TEM images of Syr/LOD@HFN after incubation in buffers with pH 6.5 for 7 days (Scale bar: 100 nm).

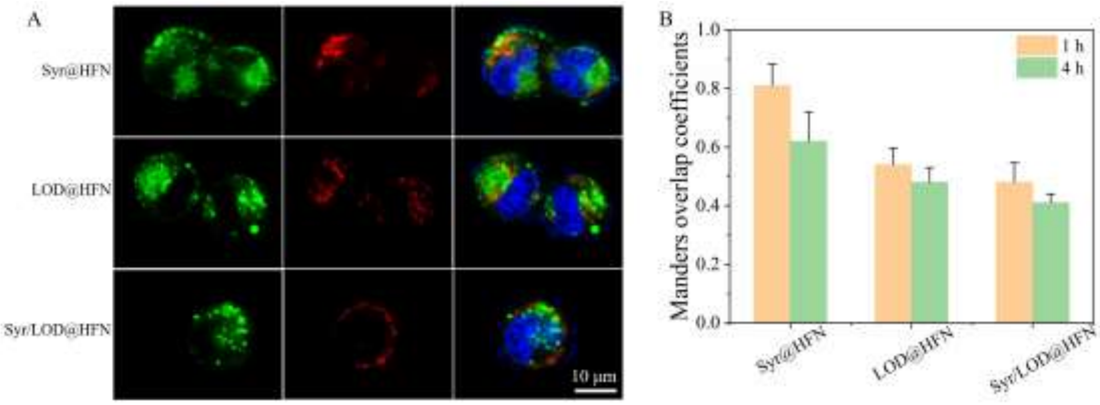

Figure S10. (A) Confocal images of Cy5.5 labelled NPs incubated with B16-F10 cells for 1 h. The lysosomes were stained with LysoTracker-Green. (B) Manders' overlap coefficient (Moc) between LysoTracker-Green and NPs-Cy5.5 signals after the nanoparticles were incubated with B16-F10 cells for 1 h or 4 h.

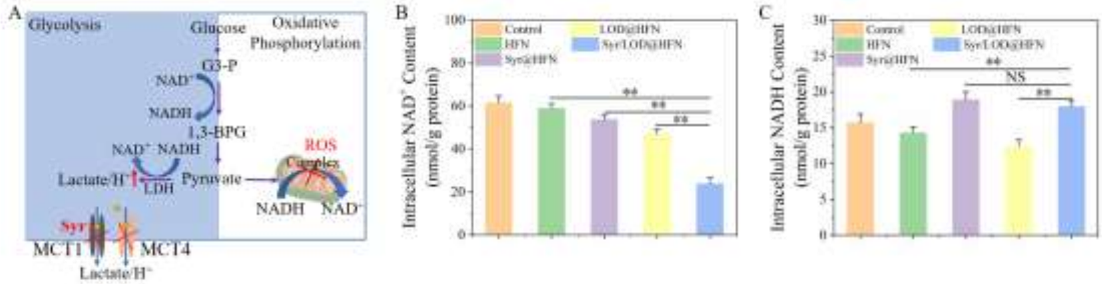

Figure S11. (A) Schematic representation of the main steps of the glycolytic pathway. (B) NAD+ and (C) NADH levels in the B16-F10 cells with NPs (100 μg/ml, pH 6.5) treatment for 24 h. (p-Values were calculated by using unpaired T-test, n = 3, $*p < 0.05$, $**p < 0.01$).

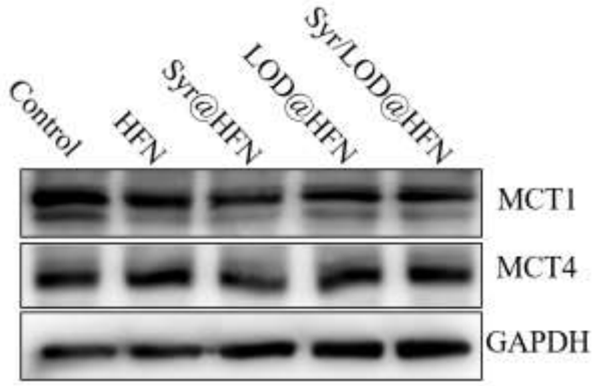

Figure S12. Western blotting for the MCT1 and MCT4 expression and their chaperone GAPDH in the B16-F10 cells after 24 h treatment by multiple nanoparticles respectively.

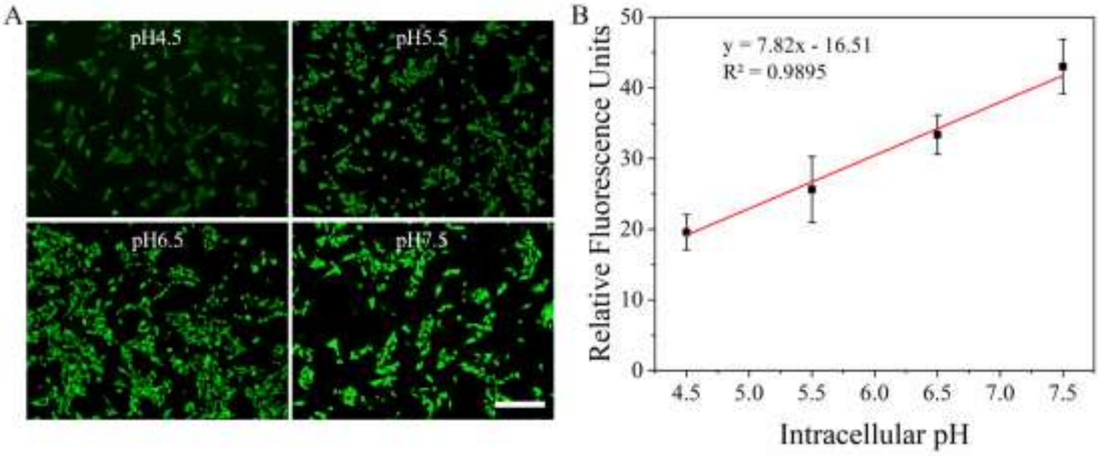

Figure S13. (A) Fluorescence images of the B16-F10 intracellular pH detected by BCECF-AM. The intracellular pH values were adjusted by an intracellular pH calibration buffer kit (P35379,Thermo Fisher Scientific). (B) The standard curve of intracellular pH linearly fitted between BCECF-AM fluorescence intensity and pH values.

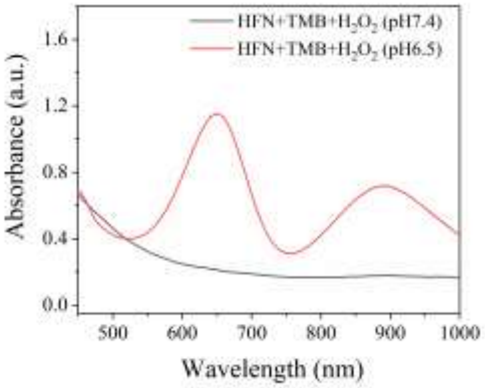

Figure S14. UV–vis absorption spectra of the catalyzed oxidation of TMB (oxTMB) as catalyzed by the HFN and H_2O_2 in the reaction buffer (pH 6.5 or pH7.4).

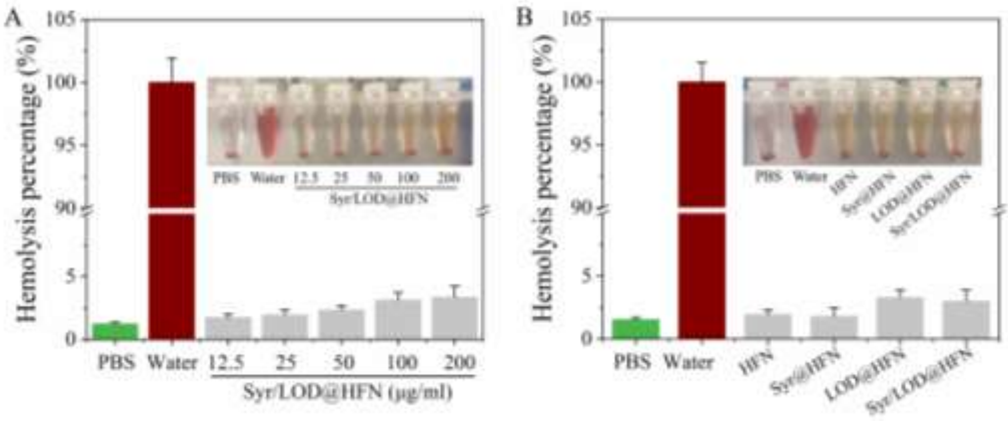

Figure S15. Hemolysis percentage of red blood cells after incubation with (A) the Syr/LOD@HFN at different concentrations and (B) various nanoparticles at concentration of 100 μg/ml for 1h .

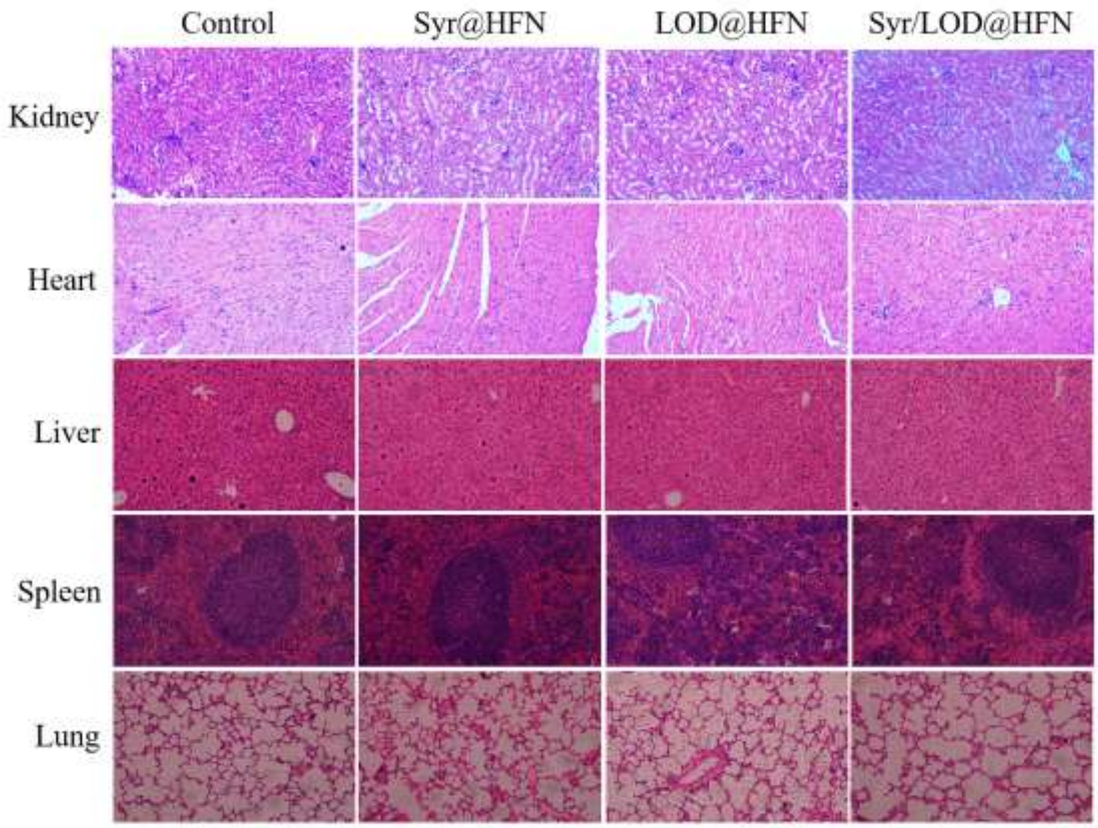

Figure S16. H&E stained slices of major organs as indicated were collected from the nude mice in group (1) Control, (2) Syr@HFN, (3) LOD@HFN and (4) Syr/LOD@HFN at day 16. No appreciable damage appeared in all major organs of the nude mice.

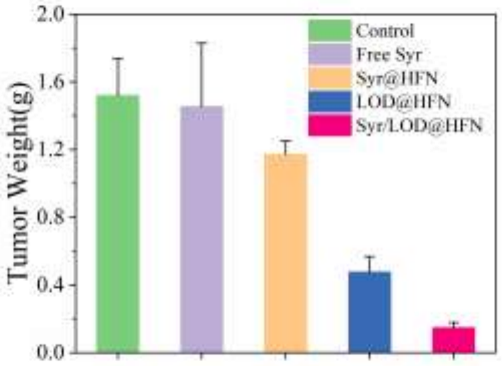

Figure S17. Tumor weight statistics of the B16-F10 tumor-bearing nude mice after different treatments.

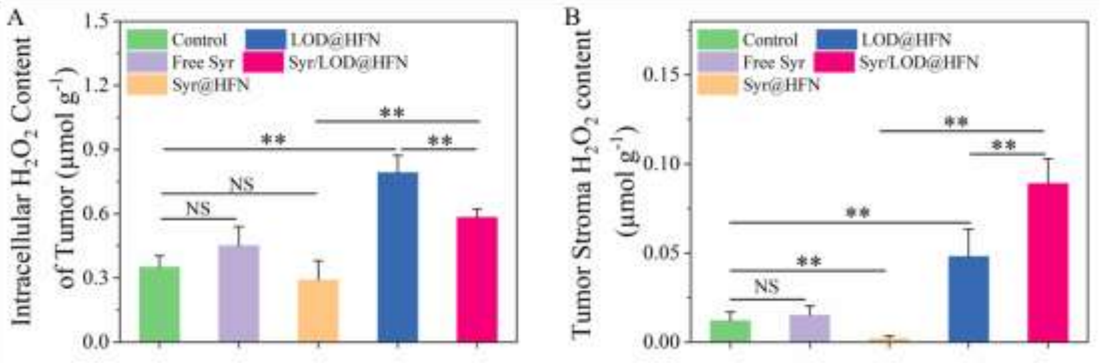

Figure S18. After treatment with multiple nanoparticles for 24 h (20 mg/kg, intravenously), H_2O_2 content in (A) tumor cells and (B) tumor stroma of nude mouse B16-F10 tumor was determined. (p-Values were calculated by using unpaired T-test, n = 4, *p < 0.05, **p < 0.01).

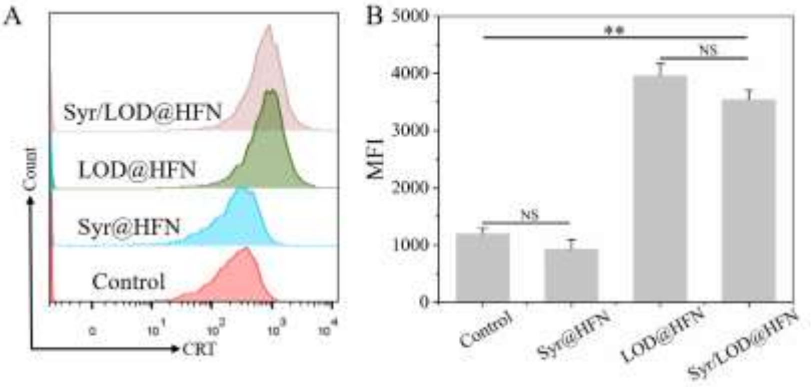

Figure S19. (A) Flow cytometry analyses and (B) MFI (mean fluorescence intensity) analyses of the representative receptors (Calreticulin) CRT^+ expression in B16-F10 cells with different treatments. (p-Values were calculated by using unpaired T-test, n = 3, *p < 0.05, **p < 0.01).

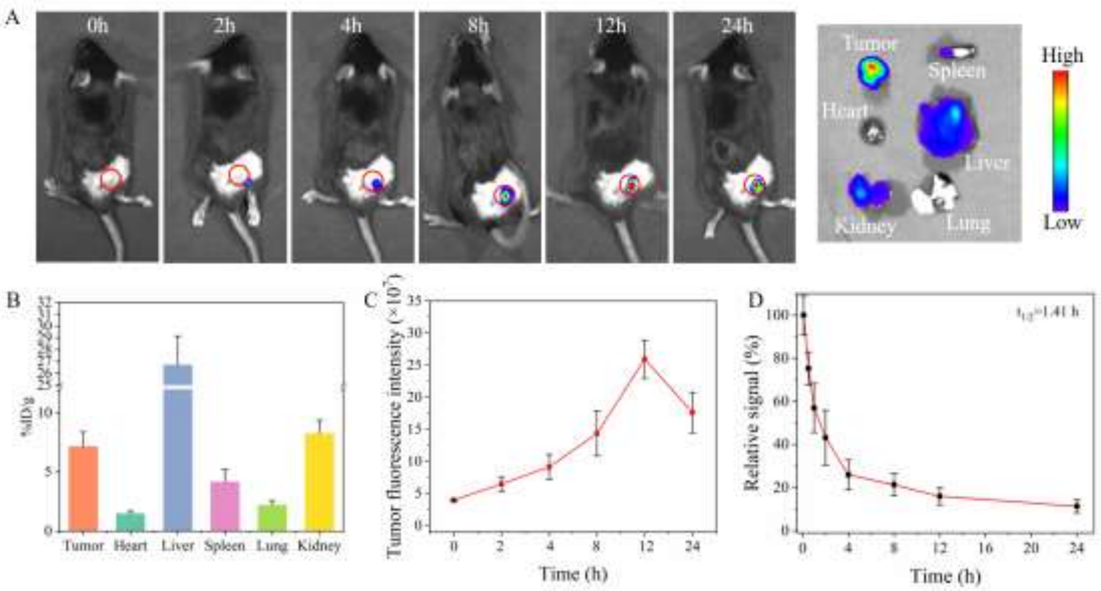

Figure S20. (A) *In-vivo* fluorescence imaging of mouse after intravenous injection of Cy5.5 labeled Syr/LOD@HFN and *ex-vivo* fluorescence imaging of mouse tumor and major organs. (B) Semiquantitative analysis of the average Cy5.5 fluorescence signals in the main organs and tumors based on the images in panel (A). (C) Semiquantitative analysis of Cy5.5 fluorescence intensity of the tumor regions according to the whole body images shown in panel (A). (D) Blood circulation profiles of the Syr/LOD@HFN by measuring the Cy5.5 fluorescence intensity of the lysed blood samples.

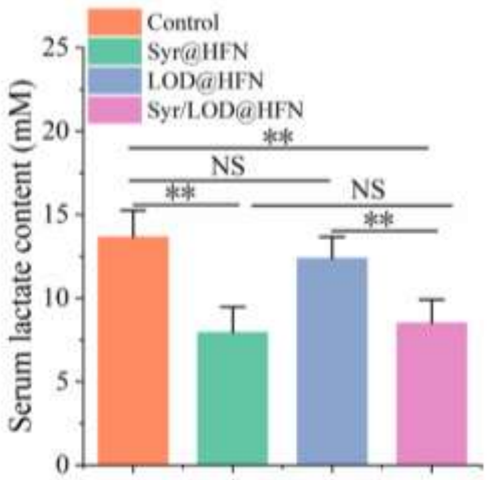

Figure S21. Lactic acid content in serum of the B16-F10 tumor-bearing C57/BL6 mice model after the treatments (n = 4). (p-Values were calculated by using unpaired T-test, n = 4, *p < 0.05, **p < 0.01).

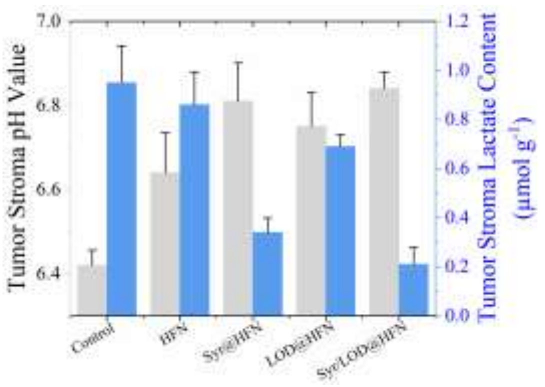

Figure S22. After 24 h treatment with different nanoparticles: (1) Control, (2) Free Syr, (3) Syr@HFN, (4) LOD@HFN, and (5) Syr/LOD@HFN, the tumor stroma lactic acid content and pH value of tissue samples from the B16-F10 tumor-bearing mice B16-F10 tumor was determined.

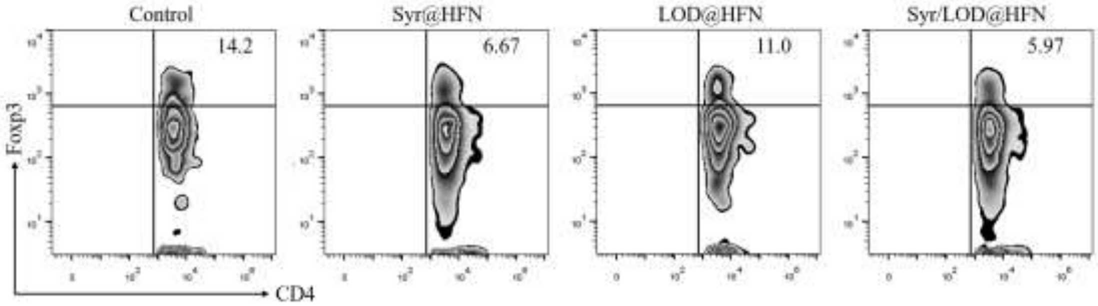

Figure S23. Representative flow cytometric zebra plots and corresponding quantification of intratumoral populations of Treg cells (CD3$^+$ CD4$^+$ Foxp3$^+$) of B16-F10 tumor-bearing mice with different treatments as indicated and analyzed (n = 4).

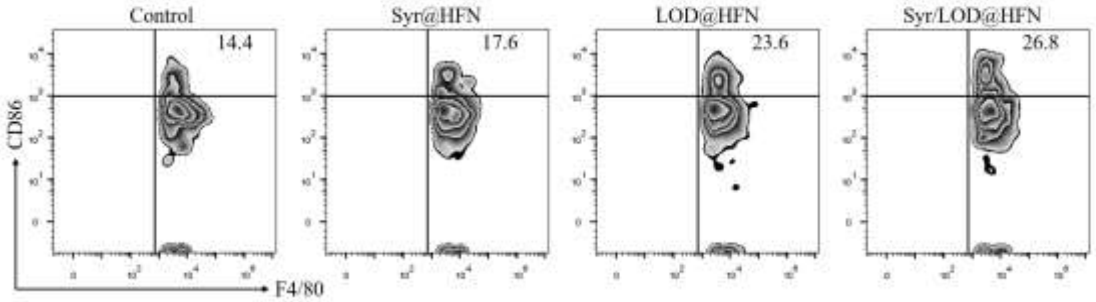

Figure S24. Representative flow cytometric zebra plots and corresponding quantification of intratumoral populations of M1 macrophages ($F4/80^+$ $CD86^+$) of B16-F10 tumor-bearing mice with different treatments as indicated and analyzed (n = 4).

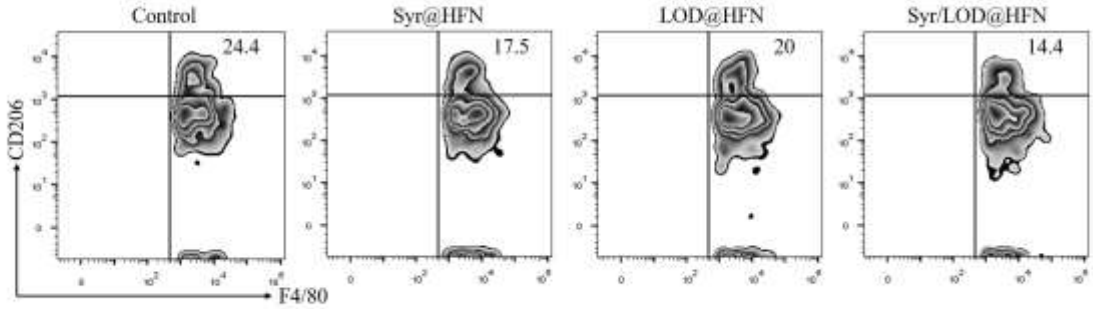

Figure S25. Representative flow cytometric zebra plots and corresponding quantification of intratumoral populations of M2 macrophages (F4/80$^+$ CD206$^+$) of B16-F10 tumor-bearing mice with different treatments as indicated and analyzed (n = 4).

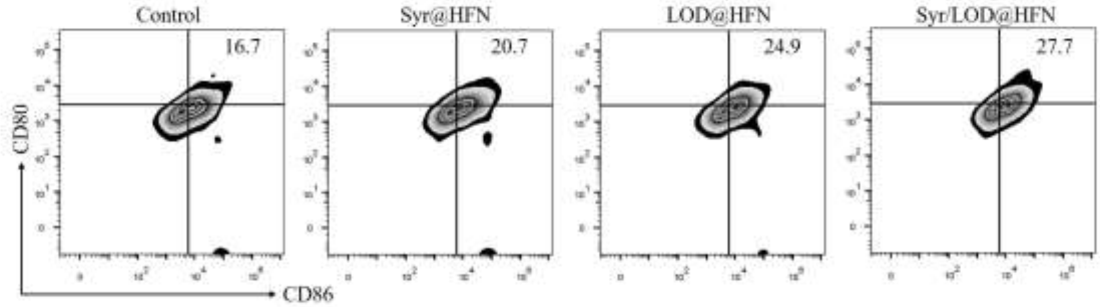

Figure S26. Representative flow cytometric zebra plots and corresponding quantification of the lymph nodes close to the populations of matured DCs ($CD11c^+$ $CD80^+$ $CD86^+$) of B16-F10 tumor-bearing mice with different treatments as indicated and analyzed (n = 4).

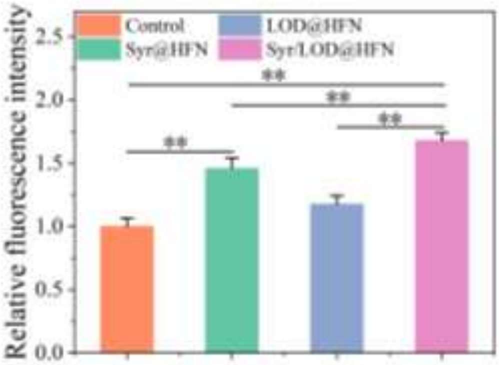

Figure S27. Quantitative analysis of fluorescence intensity of NK cells in tumor tissue (n = 4). (p-Values were calculated by using unpaired T-test, n = 4, *p < 0.05, **p < 0.01).

References

[1] X. Zhang, H. Tong, S. Liu, G. Yong, Y. Guan, *J. Mate. Chem. A.* **2013**, 25, 7488.

[2] M. Zang, W. Wang, F. Wu, K. Graveran, J. Zhang, C. Wu, *Chem. Eur. J.* **2018**, 22, 49.

[3] G. Yang, L. Xu, Y. Chao, J. Xu, X. Sun, Y. Wu, R. Peng, Z. Liu, *Nat. Commun.* **2017**, 8, 902.

[4] S. Regenspurg, A. Brand, S. Peiffer, *Geochim. Cosmochim. Ac.* **2004**, 68, 1185.

[5] F. Gao, Y. Tang, W. Liu, M. Zou, C. Huang, C. Liu, X. Zhang, *Adv. mater.* **2019**, 31(51), 1904639.

[6] a) J. Jiang, W. Wang, H. Zheng, X. Chen, X. Liu, Q. Xie, X. Cai, Z. Zhang, R. Li, *Biomaterials*. **2022**, 285, 121561; b) L. F. Ye, K. R. Chaudhary, F. Zandkarimi, A. D. Harken, C. J. Kinslow, P. S. Upadhyayula, A. Dovas, D. M. Higgins, H. Tan, Y. Zhang, M. Buonanno, T. J. C. Wang, T. K. Hei, J. N. Bruce, P. D. Canoll, S. K. Cheng, B. R. Stockwell, *ACS. Chem. biolo*. **2020**, 15.2, 469-484.
